# Gasdermin D-mediated pyroptosis is regulated by AMPK-mediated phosphorylation in tumor cells

**DOI:** 10.1038/s41419-023-06013-6

**Published:** 2023-07-26

**Authors:** Xiufeng Chu, Xiang Xiao, Guangchuan Wang, Ahmed Uosef, Xiaohua Lou, Preston Arnold, Yixuan Wang, Gangcheng Kong, Mou Wen, Laurie J. Minze, Xian C. Li

**Affiliations:** 1 Immunobiology and Transplant Science Center, Houston Methodist Hospital, Texas Medical Center, Houston, TX USA; 2 Department of Oncology, The Fifth Affiliated Hospital of Zhengzhou University, Zhengzhou, China; 3 Department of Surgery, Weill Cornell Medical College of Cornell University, New York, NY USA

**Keywords:** Immunoediting, Immunoediting

## Abstract

Gasdermin D (GSDMD) is a critical mediator of pyroptosis, which consists of a N-terminal pore-forming domain and a C-terminal autoinhibitory domain. Its cytolytic activity is sequestered by the intramolecular autoinhibitory mechanism. Upon caspase-1/11 mediated cleavage of GSDMD, the N-terminal pore-forming domain (GD-NT) is released to mediate pyroptosis. However, it remains unclear how GD-NT is regulated once it is generated. In the current study, we developed a TetOn system in which GD-NT was selectively induced in tumor cells to explore how the cytolytic activity of GD-NT is regulated. We found that the cytolytic activity of GD-NT was negatively regulated by the AMP-activated protein kinase (AMPK) and AMPK activation rendered tumor cells resistant to GD-NT-mediated pyroptosis. Mechanistically, AMPK phosphorylated GD-NT at the serine 46 (pS46-GD), which altered GD-NT oligomerization and subsequently eliminated its pore-forming ability. In our in vivo tumor model, AMPK-mediated phosphorylation abolished GD-NT-induced anti-tumor activity and resulted in an aggressive tumor growth. Thus, our data demonstrate the critical role of AMPK in negatively regulating the cytolytic activity of GD-NT. Our data also highlight an unexpected link between GSDMD-mediated pyroptosis and the AMPK signaling pathway in certain tumor cells.

## Introduction

Pyroptosis is an inflammatory cell death that is often associated with prominent tissue inflammation. Pyroptosis mainly occurs in myeloid cells upon microbial infection [1], and it also takes place in tumor cells under certain circumstances, such as the colon tumor cells treated with chemotherapy drugs [2, 3]. Several signaling cascades have been found to cause pyroptosis, and these diverse pyroptotic signals converge on the cleavage of gasdermin proteins and the subsequent release of their pore-forming N-terminal fragments [3–5].

GSDMD is the first gasdermin protein being identified as a pyroptosis mediator [5]. Structurally, GSDMD consists of a N-terminal domain and a C-terminal domain. The C-terminal domain sequesters the lipid-binding motif (β1-β2 loop) of the N-terminal domain (poring-forming or killer fragment), which therefore functions as an autoinhibitory mechanism to prevent GSDMD from interacting with the plasma membrane [6]. However, in response to canonical or noncanonical inflammasome activators, either from microbial infection or other danger signals, activated inflammatory caspases (caspase-1/4/5 in human, caspase-1/11 in mouse) cleave GSDMD to release the active pore-forming GD-NT [7, 8]. These GD-NTs self-oligomerize, insert into the plasma membrane, and then form pores to cause pyroptosis.

GSDMD-mediated pyroptosis often triggers severe tissue inflammation during infections, immune diseases, or even cancers [9–12]. Mechanisms that potentially counter GSDMD-mediated pyroptosis include recruiting the endosomal sorting complexes required for transport III (ESCRT-III) to repair the plasma membrane [13], succination by intermediates of aerobic glycolysis to prevent GSDMD cleavage by activated caspases [14]. Furthermore, Caspase-3 and Caspase-7 disable GSDMD by cleaving it at Asp87, shifting the cell fate from pyroptosis toward apoptosis [15]. Along the same line, some pathogens have devised strategies to suppress pyroptosis [16]. For example, during Enterovirus 71 infection in hand-foot-and-mouth disease (HFMD), the viral protease 3 C cleaves GSDMD and produces a noncytolytic N-terminal fragment (1-193aa) [17]. Thus, GSDMD is a potent mediator of pyroptosis and a potential target of pyroptosis regulation.

In the present study we used a TetOn system to selectively express the killer fragment GD-NT in tumor cells, asking whether GD-NT is under regulation as well as the mechanisms involved. Interestingly, we found that some tumor cells developed the resistance to GD-NT-mediated pyroptosis. Further analysis revealed that AMPK inhibited the cytolytic activity of GD-NT through phosphorylating it at the serine 46 site.

## Results

### The GD-NT-mediated pyroptosis in tumor cells is regulated by AMPK

**Fig. 1:**
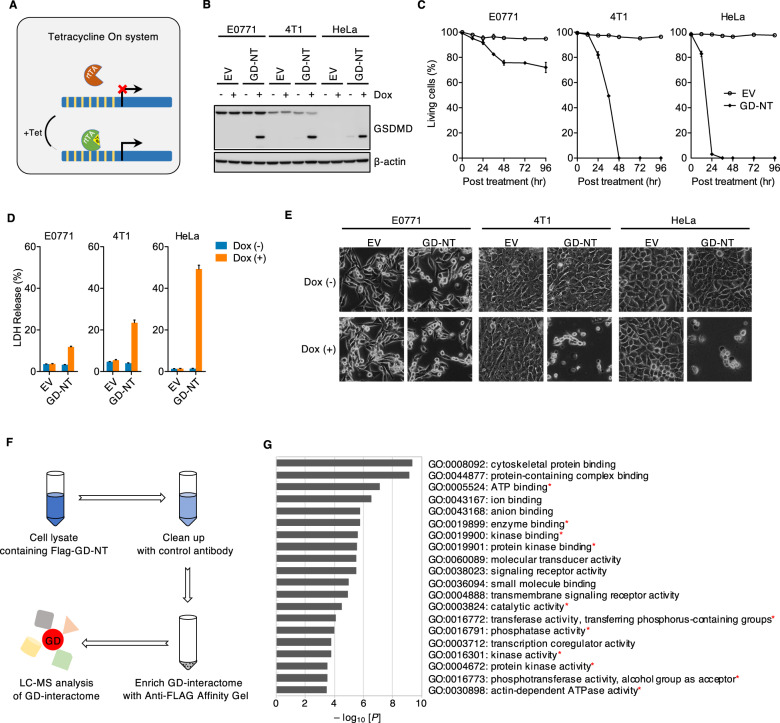
GD-NT-interacting kinases may regulate the resistance of tumor cells to GD-NT-mediated pyroptosis. **A** Tetracycline on (TetOn) system uses tetracycline (or one of its analogs like doxycycline) as a regulator of gene expression. Tetracycline-dependent promoter is created by placing a TRE upstream of a minimal promoter. TRE is 7 repeats of the tetracycline operator (tetO) sequence and is recognized by a reverse tetracycline-controlled transactivator (rtTA). In the presence of tetracycline or one of its analogs like doxycycline, rtTA will bind to tetracycline and the TRE, permitting target gene transcription. **B** Tumor cells were transduced with lentivirus to express Dox-inducible GD-NT or empty vector (EV). Cells were treated with or without Dox (2 µg/ml). GD-NT expression was detected by immunoblot (IB) analysis at 16 hours post-Dox. β-actin served as internal control. **C** Tumor cells expressing Dox-inducible GD-NT or empty vector were treated with Dox (2 µg/ml). Cell death was assessed by Trypan Blue staining at different time points. The cells that were resistant to Trypan Blue uptake under light microscopy were defined as living cells. The ratio of living cells is calculated as living cells/total cells x 100%. **D** Similar to (**C**), except that cell death was assessed with LDH-based Non-Radioactive Cytotoxicity Assay kit 24 hours post-Dox. **E** Similar to (C), except that the morphological changes were observed using phase-contrast imaging 24 hours post-Dox. **F** Anti-Flag immunoprecipitation (IP) coupled with MS analysis was performed in E0771 cells expressing Dox-inducible Flag-GD-NT to identify GD-NT-interacting proteins. Shown is a schematic workflow of LC-MS analysis of GD-NT interactome. **G**, GO enrichment analysis of GD-NT interactome with PANTHER Overrepresentation Test (molecular function). The identified proteins were acquired from the IP/LC-MS analysis (F). The interactomes of GD-NT groups and control groups were analyzed by two-tailed Student`s *t*-test (means ± s.e.m). The genes with a significant difference of fold ≥100 and *P* < 0.05 were recruited into GO analysis. GO terms were sorted based on the *P* values. Displayed are the gene ontology terms with the false discovery rate (FDR) *P* < 0.05 (Fisher’s exact test). In **C** and **D**, error bars represent the variation range of duplicated experiments. Data are representative of at least two independent experiments.

We initially found that a large number of tumor cell lines constitutively express GSDMD, but they barely expressed upstream components of the pyroptotic signaling pathway that are indispensable for GSDMD activation, such as NLRP3 (Extended Data Fig. 1A), thus making it difficult to investigate GSDMD-mediated pyroptosis in tumor cells. It is well known that GD-NT, the caspase-cleavage product of full-length GSDMD (GD-FL), is sufficient to induce pyroptosis [5] (Extended Data Fig. 1B-F). Therefore, we bypassed the upstream regulatory mechanisms and directly analyzed the cytolytic activity of GD-NT in tumor cells. To this end, we used a TetOn system in tumor cells that allowed stable expression of GD-NT upon doxycycline (Dox) treatment (Fig. 1A, B). We found that a considerable fraction of E0771 cells survived after Dox treatment. In contrast, 4T1 and HeLa cells rapidly underwent pyroptosis (Fig. 1C–E and Extended Data Fig. 1G), suggesting that E0771 cells are resistant to GD-NT-mediated pyroptosis.

To explore how E0771 cells survived the GD-NT-mediated pyroptosis, we pulled down GD-NT using immunoprecipitation (IP) assay and performed Liquid chromatography-mass spectrometry (LC-MS) to screen GD-NT-interacting proteins (Fig. 1F, G and Extended Data Fig. 1H, I). Interestingly, we found a total of 59 phospho-kinases in GD-NT interactome, which may participate in the regulation of GD-NT activity (Extended Data Table 1). Since GD-NT-mediated pyroptosis is a type of inflammatory cell death (immunogenic cell death, ICD), we narrow down our search to anti-inflammatory kinases. We found that AMPK is one of the kinases that protect cells from inflammatory injury [18, 19].

AMPK is a heterotrimeric complex composed of a catalytic α subunit, regulatory β and γ subunits. It is a highly conserved kinase from yeast to animals and plays a key role in the regulation of energy homeostasis. AMPK is activated by an elevated AMP/ATP ratio, which occurs upon cellular stress, such as heat shock, hypoxia, and ischemia [20]. Mechanistically, AMP binds two tandem domains on the gamma subunits of AMPK and triggers the phosphorylation of AMPK at Thr172 (pT172-AMPK) by the tumor suppressor kinase LKB1. The phosphorylation pT172-AMPK is required for AMPK activation, making it an ideal marker to monitor AMPK activation [21].

**Fig. 2:**
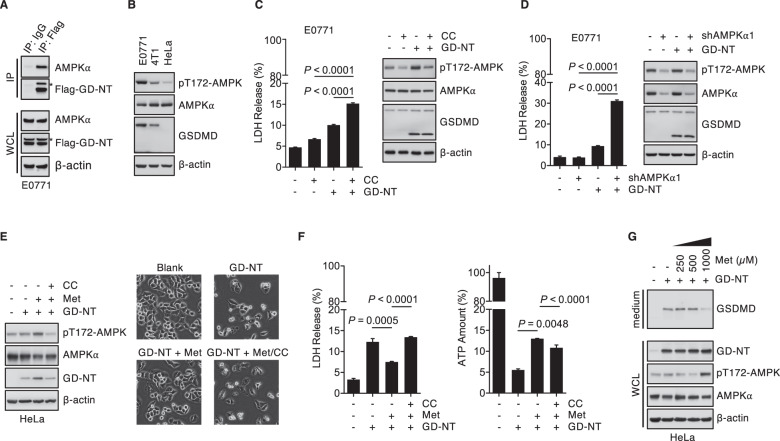
AMPK protects tumor cells from GD-NT-mediated pyroptosis. **A** GD-NT interacts with AMPKα. E0771 cells expressing Flag-GD-NT were harvested and incubated with anti-Flag M2 antibody or normal mouse IgG, followed by precipitation with Protein A/G agarose. The precipitants and whole cell lysate (WCL) were immunoblotted with indicated antibodies. **B** AMPK expression and its activation in tumor cells were assessed with IB analysis with indicated antibody. **C** Compound C sensitizes tumor cells to GD-NT-mediated pyroptosis. E0771 cells expressing Dox-inducible GD-NT were treated with Dox (2 µg/ml) and harvested for cell death assessment with LDH-based Cytotoxicity Assay (left panel) and IB analysis of WCL with indicated antibodies (right panel) 24 hours post-Dox. Compound C (CC, 5 µM) was added into the culture medium 12 hours post-Dox. **D** Inactivation of AMPK by shAMPKα1 sensitizes tumor cells to GD-NT-mediated pyroptosis. E0771 cells expressing Dox-inducible GD-NT or empty vector were modified with shAMPKα1 or scramble shRNA. Cells were treated with Dox (2 µg/ml) and harvested 36 hours post-Dox for cell death assessment with LDH-based Cytotoxicity Assay (left panel) or IB analysis of WCL with indicated antibodies (right panel). **E** Metformin protects cells from GD-NT-mediated pyroptosis. HeLa cells expressing GD-NT were treated with Dox (2 µg/ml). IB analysis of WCL was done using indicated antibodies (left panel). Phase-contrast images were taken 12 hours post-Dox (right panel). Metformin (Met, 1 mM) and/or Compound C (5 µM) were added into culture medium at the same time as Dox. **F** Similar to (**E**), except that cell death was assessed using LDH-based Cytotoxicity Assay 12 hours post-Dox (left panel), and cell survival was assessed using ATP-based cell viability Assay 16 hours post-Dox (right panel). **G** Metformin reduces the released GD-NT in the culture medium. HeLa cells expressing Dox-inducible GD-NT were treated with Dox (2 µg/ml) for 12 hours. Different concentrations of metformin or PBS were added into culture medium at the same time as Dox. Shown is IB analysis of WCL and culture medium with indicated antibodies. In **C**, **D** and **F**, differences among groups were analyzed by two-tailed Student`s *t*-test (means ± s.e.m). Error bars represent the variation range of duplicated experiments. Data are representative of at least two independent experiments.

AMPK activation not only regulates the metabolism of fatty acids and glycogen, but also regulates cell fate by participating in cell survival, apoptosis, autophagy, as well as ferroptosis [4, 22–24]. AMPK mediates such diverse effects by directly interacting with key signaling factors, such as TSC2 [24], Raptor [25], eEF2K [26], ULK1 [27, 28] and Beclin-1 [29]. We reasoned that there might be a crosstalk between AMPK and GD-NT-mediated pyroptosis. To test this possibility, we modified E0771 and U-2 OS cells to express GD-NT and GD-FL, respectively, and used IP assay to study GSDMD-AMPK interaction. The result showed that both GD-NT and GD-FL interacted with endogenous AMPK (Fig. 2A and Extended Data Fig. 2A). Consistent with the responsiveness to GD-NT-mediated pyroptosis, AMPK was highly activated in E0771 cells as compared to HeLa cells (Fig. 2B and Extended Data Fig. 2B). To further test whether AMPK affects GD-NT cytolytic activity, we downregulated AMPK activity through AMPK inhibitor Compound C or knockdown of the catalytic subunit AMPKα1 by RNA interference in E0771 cells. We found that the inactivation of AMPK sensitized these cells to GD-NT cytolytic activity and caused an increase of the lactate dehydrogenase (LDH) released in the culture medium (Fig. 2C, D and Extended Data Fig. 2C-H). In contrast, in HeLa cells with low AMPK activity, AMPK activator metformin conferred cells the resistance to GD-NT-mediated pyroptosis, causing a reduction of LDH as well as GD-NT released in the culture medium (Fig. 2E–G). These data collectively suggest that AMPK may directly regulate GD-NT-mediated pyroptosis.

### AMPK binds GD-NT to inhibit its oligomerization

**Fig. 3:**
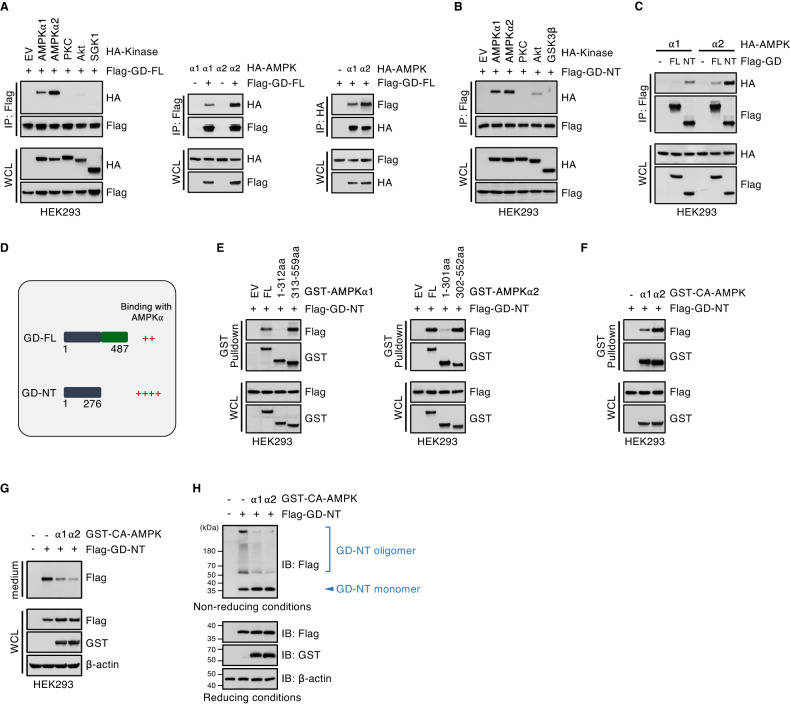
AMPKα binds to GD-NT and inhibits its oligomerization. **A** HEK293 cells were co-transfected with Flag-GD-FL and indicated HA-kinases. 24 hours later, cells were harvested for anti-Flag or anti-HA IP assay. The precipitants and WCL were immunoblotted with indicated antibodies. **B** HEK293 cells were co-transfected with Flag-GD-NT and indicated HA-kinases. 16 hours later, cells were harvested for anti-Flag assay. The precipitants and WCL were immunoblotted with indicated antibodies. **C** AMPKα1 or AMPKα2 preferably binds to GD-NT over GD-FL. Flag-GD-FL or GD-NT were co-transfected with HA-AMPKα1 or AMPKα2 into HEK293 cells. 16 hours later, Cells were harvested for anti-Flag IP assay. The precipitants and WCL were immunoblotted with indicated antibodies. **D** Schematic illustration of binding ability between AMPKα and GD-FL or GD-NT. **E** AMPKα1 or AMPKα2 binds to GD-NT through its C-terminal regulatory domain. HEK293 cells were co-transfected with Flag-GD-NT and the truncation mutations of GST-AMPKα1 (left panel) or AMPKα2 (right panel). 16 hours later, cells were harvested for GST-pulldown. The precipitants and WCL were immunoblotted with indicated antibodies. **F**, HEK293 cells were co-transfected with Flag-GD-NT and GST-constitutively active (CA)-AMPKα1 or AMPKα2. 16 hours later, cells were harvested for GST-pulldown. The precipitants and WCL were immunoblotted with indicated antibodies. **G** CA-AMPKα reduces the released GD-NT in the culture medium. Similar to (F), except that the proteins in culture medium were enriched with StrataClean beads and immunoblotted with indicated antibodies. **H** CA-AMPKα inhibits GD-NT oligomerization. Similar to (**F**), except that the cells were harvested for SDS-PAGE under both reducing and non-reducing conditions and immunoblotted with indicated antibodies. Data are representative of at least two independent experiments.

AMPKα1 and AMPKα2, two isoforms of the catalytic subunit of mammalian AMPK heterotrimeric complex, are encoded by two distinct genes. Although AMPKα1 and AMPKα2 have a comparable contribution to AMPK activity, they exhibit different substrate-targeting preference [30–32]. We further studied the interaction between GSDMD and AMPK by transfecting HEK293 cells with GSDMD and AMPKα1, AMPKα2 or other kinases. Co-immunoprecipitation (Co-IP) analysis showed that both GD-FL and GD-NT specifically interacted with AMPKα1/α2 (Fig. 3A, B). Both AMPKα1 and AMPKα2 had a binding preference for the cytolytic GD-NT, but not the noncytolytic GD-FL (Fig. 3C, D). Additionally, AMPKα1/α2 interacted with GD-NT through their C-terminal domain (Fig. 3E). These data suggest that AMPK mainly affects GD-NT activity but not that of GD-FL.

To further test whether AMPK affects the cytolytic activity of GD-NT, we transfected GD-NT with constitutively active AMPK (CA-AMPK) into HEK293 cells. Interestingly, both CA-AMPKα1 and CA-AMPKα2 bound to GD-NT and blocked its translocation into the culture medium (Fig. 3F, G). Furthermore, GD-NT oligomerization was assessed via the SDS-PAGE under non-reducing conditions (Fig. 3H), which showed a significant reduction of GD-NT oligomerization in the cells co-transfected with GD-NT and CA-AMPKα1 or CA-AMPKα2. These findings suggest that AMPK affects GD-NT cytolytic activity by suppressing its oligomerization.

### AMPK phosphorylates GD-NT at Ser46

**Fig. 4:**
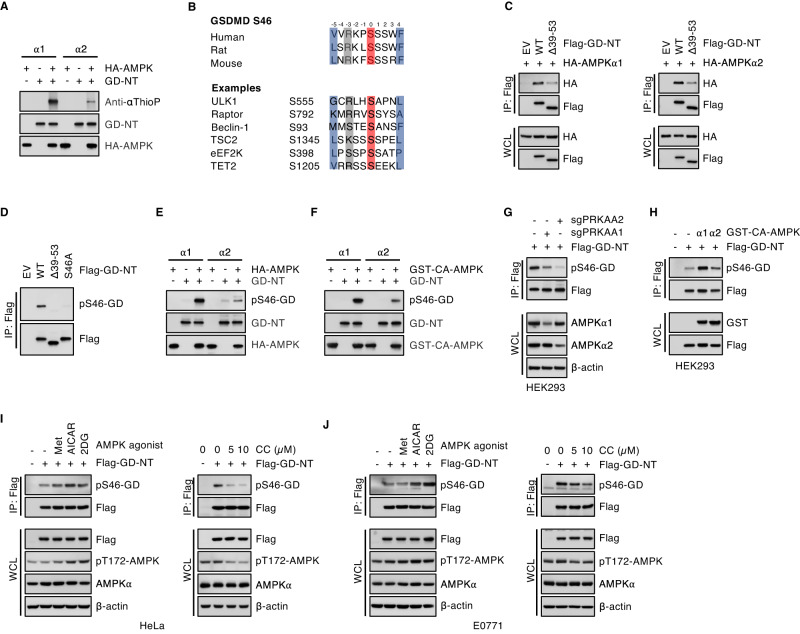
AMPK targets GSDMD-Ser46. **A** Nonradioactive in vitro AMPK kinase assay. HEK293 cells were transfected with HA-AMPKα1 or AMPKα2 and subjected to anti-HA IP assay. The precipitants were incubated with purified GST-GD-NT in a kinase reaction containing 1 mM [γS] ATP. The generated thiophosphorylation sites were alkylated with pNitrobenzyl mesylate. The products were immunoblotted with the antibody against thiophosphate ester (Anti-αThioP), HA-tag, or GST-tag. **B** Ser46 of GSDMD is an evolutionarily conserved site and matches the well-defined AMPK motif found in most substrates. Examples of classical motifs in well-known AMPK substrates are also shown. **C** HEK293 cells were co-transfected with Flag-GD-NT mutants and HA-AMPKα1 or AMPKα2. 16 hours later, cells were harvested for anti-Flag IP assay. The precipitants and WCL were immunoblotted with indicated antibodies. GD-NT-Δ39-53 is the mutant lacking the AMPK substrate motif. **D** Specificity of the generated antibody pS46-GD. HEK293 cells were transfected with Flag-GD-NT wildtype or its mutant Δ39-53 or S46A. 16 hours later, cells were harvested for anti-Flag IP assay. The precipitants were immunoblotted with indicated antibodies. **E** Similar to (**A**), except that the kinase reaction contained 200 μM regular ATP, and GD-NT phosphorylation was detected using the antibody pS46-GD. **F** Similar to (**E**), except that GST-CA-AMPKα1 or AMPKα2 were used as the phosphorylation kinase in the kinase reaction. **G** CRISPR/Cas9 technology was used to genetically disrupt the expression of AMPKα. HEK293 cells were primarily transfected with the vector expressing sgPRKAA1 and/or sgPRKAA2. After 2 days of puromycin selection, cells were further transfected with Flag-GD-NT and subjected to anti-Flag IP assay. Shown is IB analysis of anti-Flag IP and WCL with indicated antibodies. **H**, HEK293 cells were co-transfected with Flag-GD-NT and GST-CA-AMPKα1 or AMPKα2. 16 hours later, cells were harvested for anti-Flag IP assay. The precipitants and WCL were immunoblotted with indicated antibodies. **I**, HeLa cells expressing Dox-inducible Flag-GD-NT were treated with Dox (2 µg/ml) and AMPK agonist metformin (1 mM), AICAR (0.2 mM), 2-Deoxy-D-glucose (2DG, 3 mM) or AMPK antagonist Compound C (5-10 µM). 16 hours later, cells were harvested for anti-Flag IP assay. The precipitants and WCL were immunoblotted with indicated antibodies. **J** Similar to (**I**), except that the experiments were performed in E0771 cells. Data are representative of at least two independent experiments.

Next, we investigated whether AMPK regulates GD-NT through its kinase activity, we performed an in vitro non-radioactive kinase reaction in which ATPγS served as a phosphate donor. The incorporated ATPγS was alkylated to form thiophosphate ester (αThioP) and then detected using a specific antibody against αThioP. We found that both AMPKα1 and AMPKα2 phosphorylated GD-NT, and AMPKα1 exhibited a higher catalytic activity toward GD-NT than AMPKα2 (Fig. 4A).

AMPK phosphorylates consensus motif (L/M)XRXX(S/T)XXXL to regulate the activities of targeted proteins [25] (Extended Data Fig. 3A). We then utilized an algorithm to predict the AMPK substrate motif in GD-NT according to previous study [33]. The result suggested that serine 46 (Ser46) is a potential AMPK target site (Fig. 4B). Sequence alignment of GSDMD among different species showed that S46 is an evolutionarily conserved amino acid (Fig. 4B).

Ser46 locates in the β1-β2 loop, a critical motif for GD-NT binding to plasma membrane lipid [6] (Extended Data Fig. 3B). We observed that deletion of AMPK substrate motif (39-53aa) mostly abrogated GD-NT interaction with AMPKα1 or AMPKα2 (Fig. 4C). To further investigate GD-NT phosphorylation, we generated a specific antibody against the phosphorylated Ser46 (pS46-GD) and used it in the subsequent experiments to detect GD-NT phosphorylation (Extended Data Fig. 3C, D and Fig. 4D). Amazingly, both AMPKα1 (with more efficiency) and AMPKα2 catalyzed the formation of pS46-GD in our in vitro kinase assay (Fig. 4E, F and Extended Data Fig. 3E). In HEK293 cells, genetic disruption of AMPKα1 or AMPKα2 with CRISPR/Cas9 technology significantly reduced the level of pS46-GD. Conversely, overexpression of either CA-AMPKα1 or CA-AMPKα2 enhanced the level of pS46-GD (Fig. 4G, H and Extended Data Fig. 3F, G).

Next, we examined whether the commonly used AMPK-targeting chemicals influence the level of pS46-GD. HeLa cells, E0771 cells or U-2 OS cells were modified to stably express Dox-inducible Flag-GD-NT and treated with metformin, AICAR, 2-Deoxy-D-glucose or Compound C. The phosphorylation status of GD-NT in cells was analyzed by IP assay. Our results showed that pharmacological manipulation of AMPK activity significantly affected the phosphorylation pS46-GD (Fig. 4I, J and Extended Data Fig. 3H). These findings suggest that AMPK phosphorylates the Ser46 site of GD-NT.

### AMPK preferentially phosphorylates GD-NT over GD-FL

Phosphorylation is a common post-translational modification that modulates protein functions. Since AMPK preferably binds to the cytolytic GD-NT over the harmless GD-FL, we supposed that GD-NT is more likely to undergo phosphorylation than GD-FL. To test this, HEK293 cells were transfected with GD-NT, GD-FL and/or caspase-11 and subjected to IP assay. The result showed that the phosphorylation pS46-GD mainly occurred on GD-NT but not GD-FL in cells (Extended Data Fig. 4A, B). In contrast, the GD-NT generated through in vitro protease 3C-digestion was as less phosphorylated as GD-FL (Extended Data Fig. 4C-E). Therefore, we concluded that the phosphorylation pS46-GD mainly occurs after GSDMD is cleaved and activated by Caspase-11 and serves as a novel mechanism for regulating GD-NT cytolytic activity. Together with the finding that GD-NT was more phosphorylated in E0771 cells (GD-NT-resistant) than that in HeLa cells (GD-NT-sensitive) (Extended Data Fig. 5), we supposed that the phosphorylation p46-GD negatively regulates GD-NT cytolytic activity.

### Phosphorylation by AMPK abolishes GD-NT oligomerization

**Fig. 5:**
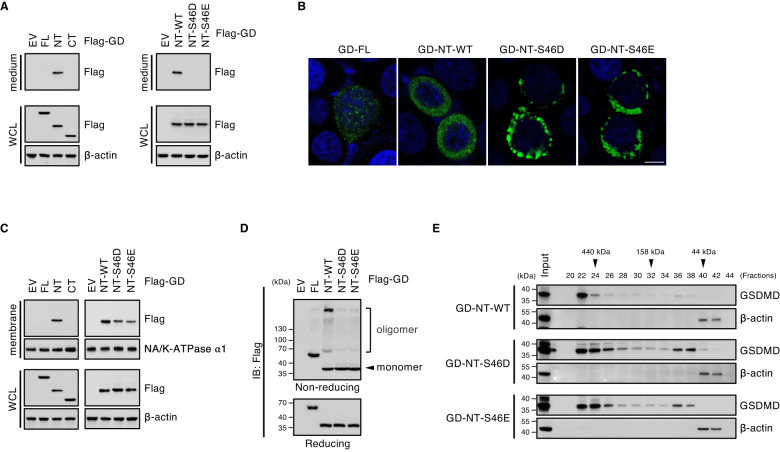
Phosphorylation affects GD-NT localization and oligomerization. **A** Detection of GD-NT in culture medium. HEK293 cells were transfected with the indicated constructs. 16 hours later, the culture medium and WCL were harvested and immunoblotted with indicated antibodies. **B** Distribution pattern of GD-NT-WT or its mutants. HeLa cells were transfected with the indicated constructs. Shown are representative confocal microscopy images of the distribution of ectopic Flag-GD-FL, GD-NT-WT, GD-NT-S46D or GD-NT-S46E (green) co-stained with DAPI (blue). **C** Subcellular localization of GD-NT-WT or its mutants. HEK293 cells were transfected with the indicated constructs. 16 hours later, cells were harvested and subjected to sample preparation using Thermo Fisher Subcellular Fractionation Kit. Membrane fractions were immunoblotted with indicated antibodies. WCL was included as an input control. **D**, Oligomerization of GD-NT. HEK293 cells were transfected with the indicated constructs. 16 hours later, cells were harvested and subjected to IB analysis with indicated antibodies under reducing or non-reducing conditions. **E** Fractionation via SEC/gel filtration. HEK293 cells were transfected with the indicated constructs. 16 hours later, cells were harvested for fractionation with SEC/gel filtration. Eluent protein fractions were immunoblotted with indicated antibodies. Input represents 10% of proteins used for SEC/gel filtration. Data are representative of at least two independent experiments.

To investigate whether the phosphorylation pS46-GD affects GD-NT cytolytic activity, we modeled the effects of AMPK-mediated phosphorylation by creating phosphomimetic mutants Ser46Asp (S46D) and Ser46Glu (S46E) and nonphosphorylatable mutant Ser46Ala (S46A). Missense3D analysis (http://www.sbg.bio.ic.ac.uk/missense3d) was used to assess the possible structural damage caused by amino acid substitution [34]. It revealed that these substitutions have no artifact structural damage and could be used for phosphorylation study (Extended Data Fig. 6A). To assess GD-NT cytolytic activity, GD-NT wild-type (GD-NT-WT) or its mutants were transfected into HEK293 cells. Culture medium and whole cell lysate were harvested for Immunoblot analysis, respectively. We found that GD-NT-S46D and GD-NT-S46E could not permeabilize the plasma membrane and translocate into the culture medium (Fig. 5A).

To answer how pS46-GD affects GD-NT cytolytic activity, the subcellular localization and oligomerization of GD-NT were investigated using different strategies [35]. Firstly, we used confocal immunofluorescence microscopy to visualize the cellular distribution of transiently expressed GD-FL, GD-NT-WT, GD-NT-S46D or GD-NT-S46E in HeLa cells. We found that, compared to GD-FL that diffusely stained the cytosol and GD-NT-WT that evenly distributed along the plasma membrane, GD-NT-S46D and GD-NT-S46E clustered near the plasma membrane (Fig. 5B). To precisely define their subcellular localization, the HEK293 cells transfected with GD-FL, GD-NT-WT or its mutants were fractionated into five compartments: soluble cytoplasmic, membrane, soluble nuclear, chromatin-bound nuclear and insoluble cytoskeletal content for immunoblot analysis (Extended Data Fig. 6B). The result showed that GD-NT-S46D and GD-NT-S46E were less distributed in membrane fraction as well as insoluble cytoskeletal fraction in comparison with GD-NT-WT (Fig. 5C and Extended Data Fig. 6C).

Next, the oligomerization of GD-NT was analyzed under non-reducing or native conditions with different strategies. HEK293 cells were transfected with GD-NT-WT, GD-NT-S46D or GD-NT-S46E and subjected to SDS-PAGE under non-reducing conditions. We found that GD-NT-S46D and GD-NT-S46E failed to form the oligomers (Fig. 5D). In addition, these transfected cells were fractionated through size exclusion chromatography (SEC)/gel filtration under native conditions and then subjected to SDS-PAGE under reducing conditions. The result revealed that GD-NT-WT formed a single huge oligomer (≥440 kDa); however, GD-NT-S46D and GD-NT-S46E distributed in a diffuse pattern (Fig. 5E). Collectively, these findings suggest that GD-NT losses the pore-forming ability when being phosphorylated at Ser46 (Extended Data Fig. 6D).

### The phosphorylated GD-NT loses its ability to mediate pyroptosis

**Fig. 6:**
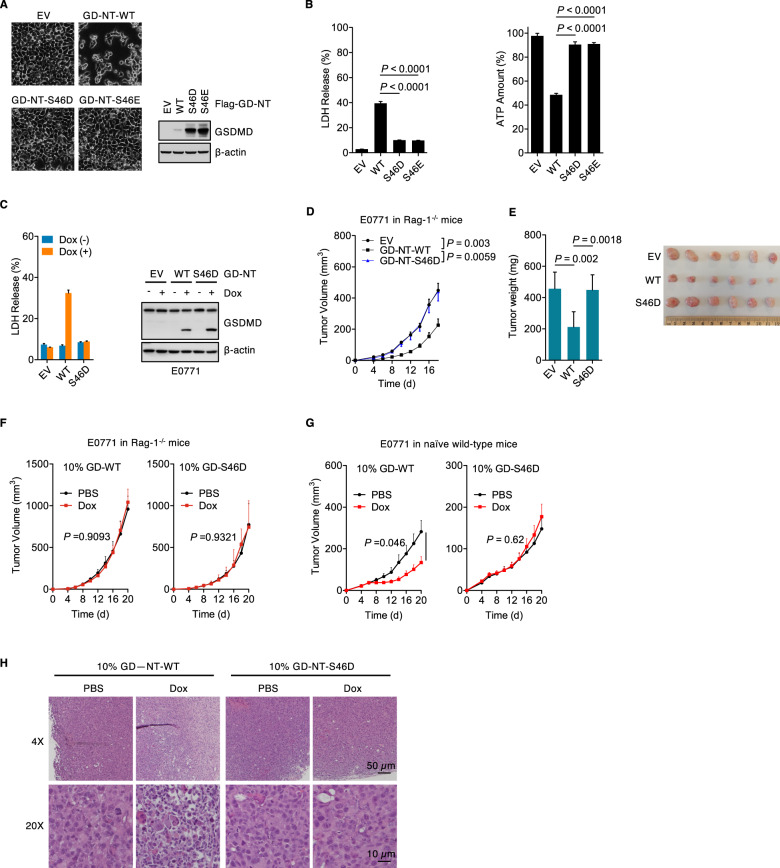
Phosphorylated GD-NT loses the ability to mediate pyroptosis and anti-tumor immunity in vivo. **A** HEK293 cells were transfected with Flag-GD-NT-WT or its mutants. 24 hours later, phase-contrast images were taken (left panel), and cells were harvested for IB analysis with indicated antibodies (right panel). **B** Similar to (**A**), except that the cells were subjected to LDH-based Cytotoxicity Assay or ATP-based Cell Viability Assay. **C** E0771 cells expressing Dox-inducible GD-NT-WT, GD-NT-S46D or empty vector were treated with or without Dox (2 µg/ml). 48 hours later, cells were harvested and subjected to LDH-based Cytotoxicity Assay. **D**, **E** E0771 tumor allograft in C57BL/6 Rag-1^-/-^ mice. E0771 cells expressing Dox-inducible GD-NT-WT or GD-NT-S46D (0.1 × 10^6^ cells per mouse) were implanted into 4^th^ mammary fat pad of Rag-1^-/-^ mice (*n* = 6 mice per group). Dox (50 mg/kg, i.p.) was administrated on day (d) 2 post-implantation and the following every other day. Tumor growth was recorded every other day and tumor weight was measured after sacrificing mice (**E**). **F**–**H**, GD-NT initiates anti-tumor immunity. 0.9 × 10^6^ of E0771 parental cells mixed with 0.1 ×10^6^ of the modified E0771 cells expressing GD-NT-WT or GD-NT-S46D were implanted into 4^th^ mammary fat pad in Rag-1^-/-^ mice (*n* = 4 mice per group) or naïve wild-type mice (*n* = 6 mice per group). Dox (50 mg/kg, i.p.) was administrated on d6 and d8 post-implantation. Tumor growth was recorded every other day (**F** and **G**). hematoxylin and eosin (H&E) staining of representative tumors at d20 was shown (H). In **B**, **C**, **D**, **E**, **F** and **G**, error bars represent the variation range of duplicated experiments. In **B** and **E**, differences among groups were analyzed by two-tailed Student`s *t*-test (means ± s.e.m). In **D**, **F** and **G**, the areas under the growth curves were compared by two-tailed Student`s *t*-test (means ± s.e.m). Data are representative of at least two independent experiments.

To measure the influence of phosphorylation on GD-NT-mediated pyroptosis, HEK293 cells were transfected with GD-NT-WT, GD-NT-S46D or GD-NT-S46E and subjected to LDH-based cell death and ATP-based cell viability assays. The results showed that GD-NT-S46D and GD-NT-S46E failed to mediate pyroptosis (Fig. 6A, B). Next, Rag-1 deficient (Rag-1^-/-^) mice and NOD.*Cg-Prkdc*^*scid*^*Il2rg*^*tm1Wjl*^/SzJ (NSG) mice, both of which lack functional T cells and B cells and exhibit the immuno-deficient phenotype, were used to test the direct influence of GD-NT-mediated pyroptosis on tumor growth in *vivo*. E0771 cells and HeLa cells stably expressing Dox-inducible GD-NT (E0771-GD-NT cells or HeLa-GD-NT cells) were inoculated in Rag-1^-/-^ mice and NSG mice, respectively. Dox was administrated via intraperitoneal injection to induce the expression of Dox-inducible genes. Consistent with the above data obtained in *vitro*, the tumors expressing GD-NT-WT had a slower growth ratio than those expressing empty vector control. In contrast, GD-NT-S46D did not efficiently suppress tumor growth (Fig. 6C–E and Extended Data Fig. 7).

Increasing evidences demonstrate that pyroptosis plays an essential role in anti-tumor immunity, and pyroptosis-induced tumor regression requires CD8^+^ T cytotoxic cells, CD4^+^ T helper cells and also Asialo-GM1^+^ nature killer cells [2, 36]. Feng Shao and his colleagues have demonstrated that 5% of total tumor cells undergoing pyroptosis is sufficient to initiate a protective antitumor immunity against cognate tumor growth and kill the other 95% of tumor cells [36]. E0771 cells have been extensively used to study the growth and development of allograft tumor in immuno-competent C57BL/6 mice. In our study, E0771 cells exhibited a significant resistance to GD-NT-mediated pyroptosis, but there were still about 20–30% of cells undergoing pyroptosis (Fig. 1C, D), which is supposed be sufficient to trigger an anti-tumor immune reaction.

To know whether GD-NT-mediated pyroptosis initiates a long-term anti-tumor immunity, immuno-competent naïve C57BL/6 mice were vaccinated with E0771-GD-NT (in the left fourth mammary fat pad and were challenged 14 days later with E0771 parental cells in the right fourth mammary fat pad. The result showed that vaccinated mice became entirely immune to the inoculation of cognate tumor cells (Extended Data Fig. 8A-E). To study the contribution of different immune cells in the formation of antitumor immunity, anti-CD4 (clone GK1.5) and anti-CD8α (clone 2.43) were used to deplete CD4^+^ and CD8^+^ T cells in vaccinated mice, respectively. Depletion efficiency was confirmed by flow cytometry. We found that depletion of CD8^+^ T cells compromised the immunity against cognate tumor cells in vaccinated mice (Extended Data Fig. 8F-H). Therefore, we conclude that GD-NT-induced anti-tumor immunity mainly relies on CD8^+^ T cytotoxic cells but not CD4^+^ T helper cells.

To further test the anti-tumor immunity induced by GD-NT-mediated pyroptosis and the influence of AMPK-mediated phosphorylation, 0.9 ×10^6^ of E0771 parental cells (90% of total inoculated cells to form tumor allograft) mixed with 0.1 × 10^6^ of E0771 GD-NT cells (10% of total inoculated cells to serve as a whole-cell vaccine) were implanted into 4^th^ mammary fat pad in Rag-1^-/-^ mice or wild-type mice. We observed a declined tumor growth and an enriched tumor-infiltrating lymphocytes in E0771-GD-WT group of wild-type mice, but not in E0771-GD-S46D group of wild-type mice or all groups of Rag-1^-/-^ mice (Fig. 6F–H). These data suggest that AMPK-mediated phosphorylation prevents GD-NT from mediating the pyroptotic cell death and the subsequent antitumor immunity.

In the last, in order to evaluate the diagnostic power of the AMPK-GSDMD axis in breast cancer, we obtained three breast cancer proteomic datasets from previous proteomic studies and performed survival analyses using the Kaplan-Meier plotter to determine the correlation between overall survival (OS) and the level of GSDMD, AMPKα, or pT172-AMPKα [37, 38]. We found that a better overall survival outcome was associated with higher expression of GSDMD and lower expression of both AMPKα or AMPKα-pT172 (Extended Data Fig. 9). GSDMD and AMPK may be proposed as new markers for biomarker-based subtyping and new targets for medical intervention in breast cancer.

## Discussion

Gasdermins are a family of pore-forming proteins. The human gasdermin family consists of GSDMA, GSDMB, GSDMC, GSDMD, GSDME and DFNB59. In the mouse, gasdermin proteins are mostly identical to human, except that it lacks GSDMB but contains different isoforms of GSDMA (A1, A2, A3) and GSDMC (C1, C2, C3, C4) [39]. Gasdermins are capable of mediating pyroptosis in immune cells and non-immune cells, such as macrophages and tumor cells. Gasdermin A- or gasdermin E-mediated pyroptosis can initiate an efficient antitumor immunity in mouse model [2, 3, 36, 40], making gasdermin a promising tool to improve the survival of cancer patients.

GSDMD is the first family member identified to mediate pyroptosis [5]. The upstream mechanisms that regulate GSDMD cleavage and activation have been extensively studied. However, it remains poorly understood whether the killer fragment GD-NT, once generated, is also subject to regulation. In this study, we demonstrated that, in selected tumor cell lines, GD-NT cytolytic activity was negatively regulated by AMPK, and AMPK activation was associated with tumor cells` resistance to GD-NT-mediated pyroptosis. We provide a novel mechanism that AMPK inhibits the pore-forming ability of GD-NT by phosphorylating GD-NT at the serine 46 site, preventing its membrane translocation and oligomerization.

We found that tumor cells exhibited different sensitivity to GD-NT-mediated pyroptosis, where E0771 cells were more resistant than other tumor cells. To understand how tumor cells resist GD-NT cytolytic activity, we analyzed GD-NT-interacting proteins in E0771 cells through LC-MS technology. GO analysis of GD-NT interactome suggested GD-NT activities are tightly related to an abundance of phosphorylation kinases. Although other kinases may also regulate GD-NT activities, we focused on the AMPK pathway, which is reported to participate in the regulation of cell death, such as apoptosis and ferroptosis [4, 24]. We found that AMPKα1 and AMPKα2 selectively bound to GD-NT and inhibited the formation of GD-NT oligomerization.

Further experiments revealed that the inhibitory effect of AMPK on GD-NT was achieved through phosphorylation at Ser46 of GD-NT. In the GD-NT crystal structure, Ser46 locates in the β1-β2 loop, a critical motif for GD-NT binding to plasma membrane lipid [6]. The phosphorylation pS46-GD hindered the normal oligomerization and localization of GD-NT on the plasma membrane. Phosphomimetic mutant GD-NT-S46D failed to mediate pyroptosis and could not trigger anti-tumor immunity. Hence, we present a substantial understanding of how tumor cells survive from GD-NT-mediated pyroptosis with the help of AMPK.

Since the initial report on the roles of GSDMD in pyroptosis in 2015, a substantial number of studies have elaborated on the structures and mechanisms of GSDMD and its homologues. The demonstration that pyroptosis can also trigger anti-tumor immunity makes GSDMD an attractive target for tumor intervention, and the clear connection between infection and inflammasome activation makes GSDMD a promising target for anti-infection treatment [41]. The development of inhibitors or agonists to regulate the pore-forming activity of GSDMD would definitely help to alleviate the detrimental effects or augment the beneficial effects of pyroptotic cell death. The link between the energy metabolism pathway (AMPK) and pyroptosis pathway (GD-NT) provides a new evidence for repurposing AMPK-targeting chemicals in GSDMD-mediated pyroptosis and cancer therapeutics. Metformin and Compound C, together with other GSDMD-targeted chemicals including Necrosulfonamide (NSA), disulfiram and Dimethylformamide (DMF) [14, 42, 43], may promote the development of pharmaceutical strategies to improve the outcomes of tumor and inflammatory diseases. Further understanding of post-cleavage regulation of gasdermins may lead us to the discovery of new interventions for pyroptosis-associated disorders.

Online Content Methods, along with any additional Extended Data display items and Source Data, and references unique to these sections, are available in the online version of the manuscript.

## Methods

### Data reporting

No statistical methods were used to predetermine sample size. The experiments were not randomized. Investigators were not blinded to allocation during experiments and outcome assessment.

### Cell lines and cell culture conditions

Raw264.7, NIH3T3, EL4, E0771, 4T1, Jurkat, HeLa, HEK293 and U-2 OS cells were purchased from ATCC. Raw264.7, NIH3T3, EL4, HeLa, HEK293 and U-2 OS cells were maintained in Dulbecco’s Modified Eagle’s Medium (DMEM) supplemented with 10% heat-inactivated fetal bovine serum (FBS), 100 U/mL penicillin and 100 µg/mL streptomycin. E0771, 4T1 and Jurkat cells were cultured in RPMI-1640 medium with the same supplements. GSDMD knockout Raw 264.7 cell line was a gift from Daniel A Bachovchin (Memorial Sloan Kettering Cancer Center). All cells were tested for mycoplasma by PCR and were authenticated by morphology only.

### Plasmids

pDB-His-MBP-mGSDMD (Addgene, 123365) was a gift from Hao Wu. pECE-HA-AMPKα1 (Addgene, 69504) and pECE-HA-AMPKα2 (Addgene, 31654) were gifts from Anne Brunet. pX462-hPRKAA1-gRNA_A (Addgene, 74374), pX462-hPRKAA1-gRNA_B (Addgene, 74375), pX462-hPRKAA2-gRNA_A (Addgene, 74376), pX462-hPRKAA2-gRNA_B (Addgene, 74377) were gifts from Reuben Shaw. pTRIPZ-shNS (Addgene, 127696) was a gift from Sandra Demaria. LT3GEPIR was a gift from Johannes Zuber (Addgene,111177). The vectors expressing mouse GSDMD, human AMPKα1, human AMPKα2, human PKCα, human Akt1, human SGK1 and human GSK3β were generated by the standard PCR cloning strategy. Truncation mutation or point mutation plasmids were generated using QuickChange Primer Design Program and mutagenesis kit (Agilent Technologies). Constitutively active AMPK α1 (1-312aa) and AMPKα2 (1-301aa) were generated as described previously [44]. All plasmids were verified by DNA sequencing and IB analysis.

### Reagent and antibodies

LPS O111:B4 (L2630), Doxycycline (D3447), 2-Deoxy-D-glucose (D8375) and compound C (P5499) were obtained from Sigma-Aldrich. Subcellular protein fractionation kit (78840) was obtained from Thermo Fisher. Metformin (S1950) and AICAR (S1802) were obtained from Selleck Chemicals. ATP (tlrl-atpl) was obtained from InvivoGen. StrataClean resin (400714) and chemical competent cells (200315) were obtained from Agilent.

For immunoblot, AMPKα1(#2795), AMPKα2 (#2757), AMPKα1/2 (#5831) and pT172-AMPK (#2535) were purchased from Cell Signaling Technology. Anti-Flag (F1804), anti-Flag (F7425) and anti-HA (H6908) were obtained from Sigma-Aldrich. Anti-GSDMD (ab219800) and anti-Caspase-1 (ab108362) were obtained from Abcam. Anti-β-Actin (sc-47778), anti-GST (sc-138) and anti-Na/K-ATPase α1 (sc-21712) were purchased from Santa Cruz Biotechnology. Anti-IL18 (A1115) was obtained from Abclonal Technology. phospho-GSDMD S46 was generated by Abclonal Technology.

For FCAS analysis, CD45-APC-Cy7(clone 30-F11), CD3-FITC (clone 145-2C11), CD8-PerCP-Cy5.5 (clone 53-6.7) and CD4-APC (clone RM4-5) were obtained from BioLegend.

### Stable cell lines

Lentivirus was produced in HEK293 cells by transfection of the lentiviral vector with psPAX2 (Addgene) and pMD2.G (Addgene). Lentiviral supernatants were collected, filtered through 0.45-µm filters, and used to transduce E0771, 4T1 and HeLa cells. Polybrene infection/transfection reagent (Millipore, 10 µg/ml) was added to increase the efficiency of lentiviral infection. After 2 days of transduction, puromycin (Sigma, 2 µg/ml) was added to select the transduced cells. Empty lentiviral vectors were used to generate control cells. The expression of relevant genes in stable cell lines was verified by immunoblot.

### CRISPR-Cas9 knockout cells

AMPK double knockouts (DKO) were generated using the Cas9 nickase strategy as described [45]. Briefly, each duplex of guide RNAs (gRNA) pair targeting the exon 1 of human PRKAAA1 or PRKAA2 was cloned into pX462 vector [44]. HEK293 cells were transfected with one pair to generate single AMPK α1 or α2 knockout or transfected with both pairs together to generate AMPK α1/α2 double knock (DKO). After 2 days of puromycin selection, knockout cells (pool) were directly used for signaling analysis or further single cell cloning. Individual clones were screened by immunoblot and an AMPK DKO clone was used to reconstitute either α1 or α2 to assess their effects on GD-NT.

### LC-MS analysis of GD-NT interactome

The IP’ed beads were resolved on NuPAGE 10% Bis-Tris Gel with MOPS running buffer (Life Technologies). The eluted proteins were visualized with Coomassie Brilliant blue-stain, excised into gel pieces and in-gel digested with trypsin. The LC-MS/MS analysis was carried out using nanoLC1200 system coupled to Orbitrap Fusion Lumos mass spectrometer (Thermo Scientific, San Jose, CA). The peptides were loaded on a two-column setup with precolumn (2 cm × 100 µmI.D.) and analytical column (5 cm × 150 µmI.D.) filled with Reprosil-Pur Basic C18 (1.9 µm, Dr. Maisch GmbH, Germany). The peptide elution was done using a discontinuous gradient of 90% acetonitrile buffer (B) in 0.1% formic acid (5-28% B, 750 nl/min: 75 min gradient). The MS instrument was operated in data dependent mode with MS1 acquisition in Orbitrap (120000 resolution, AGC 5e5, 50 ms injection time) followed by MS2 in Ion Trap (Rapid Scan, HCD 32%, AGC 5e4). The MS raw data were searched using Proteome Discoverer 2.1 software (Thermo Scientific, San Jose, CA) with Mascot algorithm against mouse or human NCBI RefSeq database updated 2020_0324. The precursor ion tolerance and product ion tolerance were set to 20 ppm and 0.5 Da, respectively. Maximum cleavage of 2 with Trypsin enzyme, dynamic modification of Oxidation on methionine, Protein N-term acetylation and Destreak on cysteine was allowed. The peptides identified from the mascot result file were validated with a 5% false discover rate (FDR). The gene product inference and quantification were done with a label-free iBAQ approach using ‘gpGrouper’ algorithm [46].

### Immunoblots and immunoprecipitation

Cells were lysed in EBC buffer (50 mM Tris pH 7.5, 120 mM NaCl, 0.5% NP-40) supplemented with protease inhibitors (A32953, Thermo Fisher) and phosphatase Inhibitors (B15002, Bimake). The protein concentrations of lysates were measured using the Beckman Coulter DU-800 spectrophotometer and the Bio-Rad protein assay reagent. Same amounts of whole cell lysates were resolved by SDS-PAGE and immunoblotted with indicated antibodies. For immunoprecipitation, cell lysates containing 1 mg of total proteins were incubated with anti-Flag agarose (A2220, Sigma) or anti-HA Agarose (A2095, Sigma) for 4 hours at 4 °C. Precipitants were washed three times with EBC buffer and resolved by SDS-PAGE followed by immunoblot analysis with indicated antibodies.

### Protein enrichment from the culture medium or peritoneal fluid

To enrich the proteins in the culture medium, 1 ml of culture medium was centrifuged at 14,000 x *g* for 10 min at 4 °C to remove cellular debris. 10 µl StrataClean resin (400714, Agilent) was then added for 1-hour incubation on a rotator at 4 °C. The supernatant was removed by centrifuge. The resin was harvested and suspended in 50 µl 2 x loading buffer for immunoblot. In septic mice model, the proteins in flushed peritoneal fluid were also enriched in the same way.

### Size exclusion chromatography (SEC)

SEC was performed using an AKTA Purifier system (GE Healthcare, Buckinghamshire, England). A HiLoad 16/600 Superdex 200 column (GE Healthcare) was equilibrated with cell lysis buffer. The column was calibrated using a gel filtration calibration kit (GE Healthcare). Each standard protein was dissolved in cell lysis buffer and chromatographed on the column separately. The filtered protein samples were then fractionated on the column (1.0 ml/min; 2 ml/fraction). For immunoblot analysis, proteins in fractionated eluent were enriched with 10 µl StrataClean resin and harvested in 30 µl of 2 x loading buffer.

### AMPK in vitro kinase assays

In [γS] ATP-containing kinase reaction, 1 μg of GST-GD-NT purified from HEK293 cells transfected with the indicated constructs, 300 ng of AMPK complex prepared from the HEK293 cells transfected with the indicated constructs, 1 mM [γS] ATP (ab138911, Abcam) were added in kinase reaction buffer (50 mM Tris (pH 7.5), 1 μM MnCl_2_, 2 mM dithiothreitol (DTT)). After 30 min of incubation at 30 °C, the reaction was subsequently stopped by adding in 0.1 mM EDTA, and further reacted for another 1 h by adding pNitrobenzyl mesylate (ab138910, Abcam) to alkylate the thiophosphorylation site on the substrates. The reaction was stopped by adding SDS loading buffer and resolved by SDS–PAGE. Phosphorylation of GD-NT was detected using anti-Thiophosphate ester (ab92570, Abcam).

In regular ATP-containing kinase reaction, 1 μg of GST-GD-NT purified from HEK293 cells transfected with the indicated constructs, 300 ng of AMPK complex prepared from the HEK293 cells transfected with the indicated constructs or commercially obtained from Sigma (14-840), 200 μM regular ATP were added in kinase reaction buffer (50 mM Tris (pH 7.5), 1 μM MnCl_2_, 2 mM dithiothreitol (DTT)). After 30 minutes of incubation at 30 °C, the reaction was stopped by adding SDS loading buffer and resolved by SDS–PAGE. GD-NT phosphorylation was detected using the antibody pS46-GD.

### Cytotoxicity assay and cell viability assay

Cell death and cell viability were performed using Non-Radioactive Cytotoxicity Assay kit (G1780, Promega) and CellTiter-Glo Luminescent Cell Viability Assay kit (G7571, Promega), respectively. Briefly, 5 ×10^3^ cells were cultured in 96-well plates with Opaque wall. At the desired time points, cell death was determined by titrating the amount of lactate dehydrogenase released into the culture medium, and cell viability was determined by the ATP levels within cells, according to the manufacturer’s instructions.

### Mouse studies

All procedures were conducted in compliance with American guidelines for the care and use of laboratory animals and were approved by the Houston Methodist Animal Care Committee in accordance with institutional animal care and use guidelines. Female wild-type C57BL/6 mice, Rag-1^-/-^ mice and NSG mice (6-8 weeks old) were purchased from Jackson Laboratories. All mice were housed in the Houston Methodist Animal Facility. Before inoculation, cell viability was determined using trypan blue exclusion test (minimum of 98% cell viability). For tumor-challenge experiments in immuno-deficient mice, 0.1 ×10^6^ of E0771 cells or 0.5 ×10^6^ of HeLa cells that express empty vector, GD-NT-WT, GD-NT-S46D or GD-NT-S46A were injected subcutaneously into the right flank of NSG mice. To vaccinate mice, 1.0 ×10^6^ of E0771-EV cells or E0771-GD-NT cells were injected into in the left fourth mammary fat pad. For tumor-challenge experiments in vaccinated mice, 1.0 ×10^6^ of E0771 parental cells were injected into in the right fourth mammary fat pad. In another independent investigation, anti-tumor immunity was initiated by implanting the combination of 0.9 ×10^6^ of E0771 parental cells and 0.1 ×10^6^ of the modified E0771 cells expressing GD-NT-WT or GD-NT-S46D into 4^th^ mammary fat pad. Doxycycline (50 mg/kg, i.p.) was given via Intraperitoneal injection at indicated timepoints to induce the expression of GD-NT-WT or GD-NT-S46D. Tumor growth was monitored every other day. Tumor volume (mm^3^) is calculated via the “(W x W x L) / 2” formula, where L is the longest diameter and W is the shortest diameter. Necropsy and tumor collection were performed at the end of tumor size recording. For immune cell depletion, each mouse was intraperitoneally administered 200 μg of anti-CD4 (clone GK1.5, BioXCell), anti-CD8α (clone 2.43, BioXCell) or isotype control (clone LTF-2, BioXCell) antibody on day -4, -3 -2 of tumor challenge. To verify the depletion efficiency, the percentage of CD4^+^ or CD8^+^ T cells was determined by flow cytometry on day 0 in blood, spleen and skin.

### Flow cytometry

Single-cell suspensions were first incubated with viability dyes (Zombie Aqua; Biolegend) to identify dead cells and with an anti-FcγRII/III antibody (2.4G2; BD Bioscience) to block nonspecific antibody binding. After washing, the cells were incubated with mixtures of fluorescently labeled antibodies for 20 min at 4 °C. Samples were acquired using an LSRFortessa™ or LSR II flow cytometer (BD Bioscience) and analyzed using FlowJo software (Tree Star).

### Statistical analysis

Student`s *t*-test was used to determine the differences between the two groups. Differences between tumor growth curves were compared by calculating the area-under-curve values for each sample and then comparing different groups using Student’s *t*-test. The results are presented as the mean and standard error of the mean (SEM). Statistical significance was assigned to *P* < 0.5%. Tumor-free survival and Kaplan-Meier analysis were performed using GraphPad Prism Version 5.04 for Windows.

### Reporting summary

Further information on research design is available in the Research Reporting Summary linked to this paper.

## Supplementary information

Supplementary Materials for Gasdermin D-mediated pyroptosis is regulated by AMPK-mediated phosphorylation in tumor cells

Reproducibility checklist

CDDis Uncropped original western blot

## Data Availability

All relevant data are available in the Source Data (for Figs. 1–6 and Extended Data Fig. 1-9) or supplementary information associated with this paper.
