# Supplementary material for: Gasdermin D-mediated pyroptosis is regulated by AMPK-mediated phosphorylation in tumor cells
Source: Cell Death Dis. 2023 Jul 26;14(7):469. doi: 10.1038/s41419-023-06013-6 (PMC10372026; doi:10.1038/s41419-023-06013-6)
Supplement: Supplementary file 1 — Supplementary Materials for Gasdermin D-mediated pyroptosis is regulated by AMPK-mediated phosphorylation in tumor cells [file 41419_2023_6013_MOESM1_ESM.pdf]

Supplementary Materials for

Gasdermin D-mediated pyroptosis is regulated by AMPK-mediated phosphorylation in tumor cells

Xiufeng Chu[1,2], Xiang Xiao[1], Guangchuan Wang[1], Ahmed Uosef[1], Xiaohua Lou[1], Preston Arnold[1], Yixuan Wang[1], Gangcheng Kong[1], Mou Wen[1], Laurie J. Minze[1], Xian C. Li[1,3*]

*Corresponding author. Email: xxcli@houstonmethodist.org

This file includes:

Extended Data Fig. 1 to 9

Extended Data Table 1

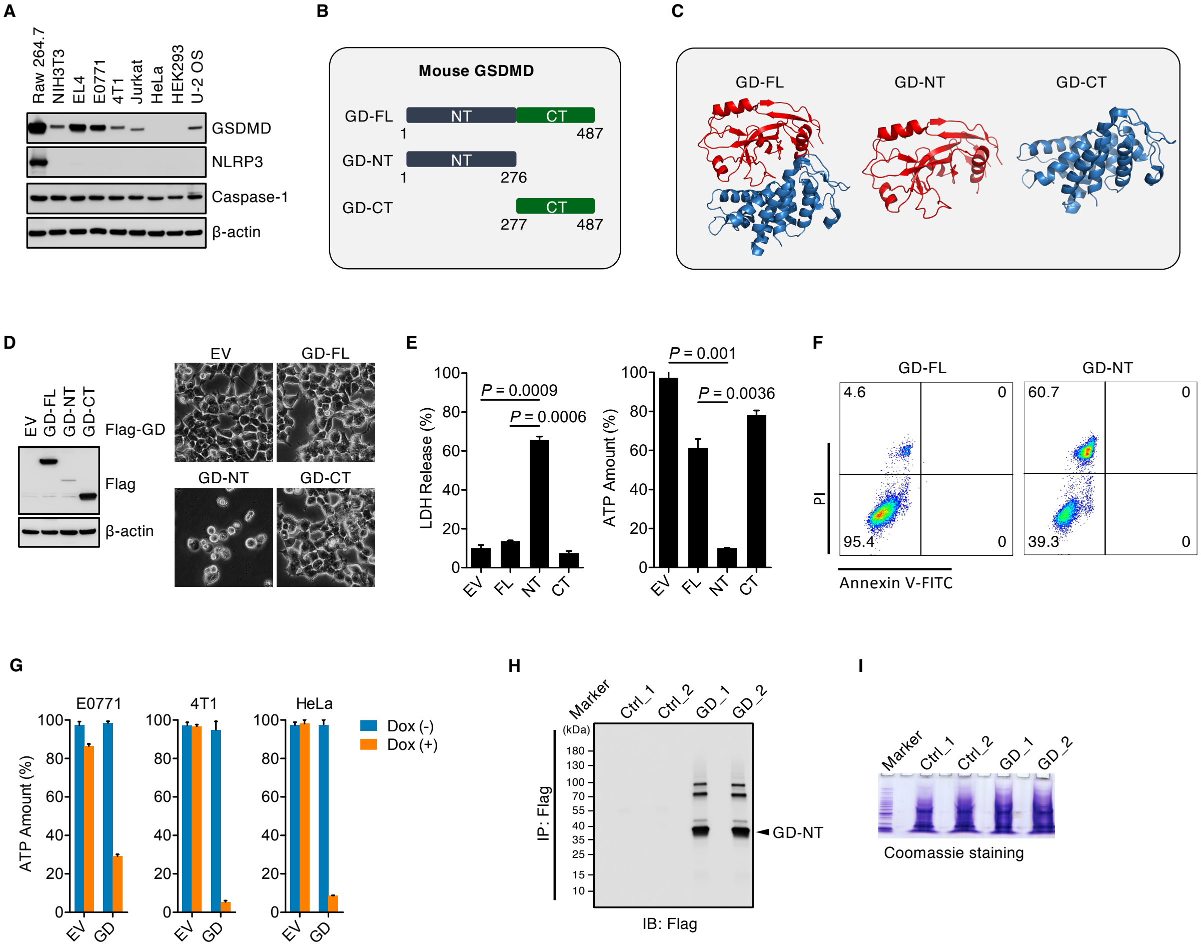

Extended Data Fig. 1 I GD-NT mediates pyroptotic cell death.

A, GSDMD and its upstream components in different cells were assessed with immunoblot (IB) analysis with indicated antibodies. β-actin served as internal control.

B, Schematic illustration depicts the structure of mouse GSDMD.

C, Structure of mouse GSDMD was downloaded from RCSB PDB (PDB code: 6N9N). PyMOL was used to generate structure model of GD-FL, GD-NT and GD-CT.

D, GD-NT-mediated pyroptosis. HEK293 cells were transfected with the construct expressing GD-FL, GD-NT, GD-CT or empty vector (EV). 24 hours later, cells were harvested for further analysis. Shown are IB analysis of WCL (left panel) and phase-contrast images (right panel).

E, Similar to (D), except that the cells were subjected to LDH-based Cytotoxicity Assay (left panel) or ATP-based Cell Viability Assay (right panel).

F, Similar to (D), except that the cells were harvested for FACS analysis using Annexin V-FITC/PI Double staining Kit 16 hours post-transfection. Dots in lower right quadrant represented apoptotic cells.

G, Tumor cells expressing Dox-inducible GD-NT or empty vector were treated with Dox (2 μg/ml). Cell survival was assessed using ATP-based cell viability Assay 24 hours post-Dox.

H, IB analysis of IP samples with indicated antibody were performed as quality control prior to LC-MS analysis.

I, Coomassie blue staining gel of IP samples were performed as quality control prior LC-MC analysis.

In E and G, Error bars represent variation range of duplicated experiments. In E, differences among groups were analyzed by two-tailed Student`s *t*-test (means ± s.e.m). Data are representative of at least two independent experiments.

Extended Data Table 1 l GD-NT interacting kinases. Anti-Flag immunoprecipitation (IP) coupled with LC-MS analysis was performed to identify GD-NT-interacting proteins in E0771 cells. Shown are the kinase-encoding genes that are 5-fold richer in GD-NT group over control group.

GeneSymbol	Function	Signaling pathway	Fold changes Log(GD-NT/ctrl)
Akt2	intracellular kinase	Akt/mTOR	1047
Atm	serine/threonine protein kinase	DNA damage check point	2.78
Axl	receptor tyrosine kinase	multifunctional enzyme involved in many biological processes	1.94
Blk	non-receptor tyrosine kinase	B-lymphocyte signaling	8
Camk2d	calcium/calmodulin-dependent protein kinase	Ca^{2+} homeostatis	204
Camk2g	calcium/calmodulin-dependent protein kinase	Ca^{2+} homeostatis	33
Cdc42bpb	serine/threonine-protein kinase	cytoskeleton reorganization and cell migration	5
Cdk1	cyclin-dependent kinase	cell cycle progression	7
Cdk2	cyclin-dependent kinase	cell cycle progression	3.61
Cdk9	cyclin-dependent kinase	cell cycle progression	24
Cdk11b	cyclin-dependent kinase	cell cycle progression	2.11
Cdk12	cyclin-dependent kinase	cell cycle progression	24
Cdk20	cyclin-dependent kinase	cell cycle progression	1.39
Csk	non-receptor tyrosine-protein kinase	cell migration and immune response	7.49
Csnk1a1	casein kinases	beta-catenin destruction complex assembly	34
Csnk1d	casein kinases	beta-catenin destruction complex assembly	2.46
Csnk2a1	casein kinases	beta-catenin destruction complex assembly	5
Csnk2a2	casein kinases	beta-catenin destruction complex assembly	12
Dapk3	death-associated protein kinase	apoptosis	7.68
Dyrk1a	dual-specificity kinase	cell proliferation	798
Eif2ak3	metabolic-stress sensing protein kinase	stress response	12316
Fgfr1	tyrosine-protein kinase	cell-surface receptor for fibroblast growth factors	1.39
Fgfr2	tyrosine-protein kinase	cell-surface receptor for fibroblast growth factors	1.39
Fgfr3	tyrosine-protein kinase	cell-surface receptor for fibroblast growth factors	1.39
Fgfr4	tyrosine-protein kinase	cell-surface receptor for fibroblast growth factors	1.39
Fgr	non-receptor tyrosine-protein kinase	signaling for cell surface non-kinase receptors	1.39
Fyn	non-receptor tyrosine-protein kinase	multifunctional enzyme involved in many biological processes	1.39
Gsk3b	glycogen synthase kinase	glucose homeostasis	8.51
Hck	non-receptor tyrosine-protein kinase	innate immune responses	1.39
Ick	serine/threonine-protein kinase	ciliogenesis	1.39
Jak1	non-receptor tyrosine-protein kinase	IFN-alpha/beta/gamma signal pathway	64517
Lck	non-receptor tyrosine-protein kinase	maturation and function of T-cell	1.39
Lyn	non-receptor tyrosine-protein kinase	B cell homeostasis	1.39
Mak	serine/threonine-protein kinase	regulation of ciliary length	1.39
Map3k4	MAP kinase	cell proliferation and differentiation	3.76
Map3k7	MAP kinase	cell proliferation and differentiation	240
Map4k5	MAP kinase	cell proliferation and differentiation	2.76
Mapk1	MAP kinase	cell proliferation and differentiation	2
Mapk6	MAP kinase	cell proliferation and differentiation	1548
Mapk7	MAP kinase	cell proliferation and differentiation	8.07
Mapkapk5	MAP kinase	cell proliferation and differentiation	7.14
Mark3	serine/threonine-protein kinase	microtubule cytoskeleton organization	2
Mast3	serine/threonine-protein kinase	microtubule cytoskeleton organization	8.96
Pdgfra	receptor Tyrosine-protein kinase	cell proliferation and cell migration	3.26
Pkn1	PKC-related serine/threonine-protein kinase	multifunctional enzyme involved in many biological processes	2.68
Plk1	serine/threonine-protein kinase	cell cycle	13
Prkaa1	AMP-activated protein kinase (AMPK)	energy metabolism, autophagy	5
Prkacb	cAMP-dependent protein kinase (PKA)	cAMP-dependent signaling	1.09
Prkcd	serine/threonine-protein kinase (PKC)	multifunctional enzyme involved in many biological processes	1.13
Prkci	serine/threonine-protein kinase (PKC)	multifunctional enzyme involved in many biological processes	5
Prpf4b	serine/threonine-protein kinase	pre-mRNA splicing	2.25
Riok1	serine/threonine-protein kinase	maturation of SSU-rRNA	339
Ripk3	serine/threonine-protein kinase	necroptosis and apoptosis	0.57
Rps6ka4	serine/threonine-protein kinase	inflammatory response, Stress response	33
Stk3	serine/threonine-protein kinase	stress-activated, pro-apoptotic kinase	4.25
Stk38	serine/threonine-protein kinase	negative regulator of MAP3K1/2 signaling	225682
Stk38l	serine/threonine-protein kinase	differentiating and mature neuronal cells	38901
Ulk1	serine/threonine-protein kinase	autophagy	6.07
Yes1	non-receptor protein tyrosine kinase	multifunctional enzyme involved in many biological processes	1.39

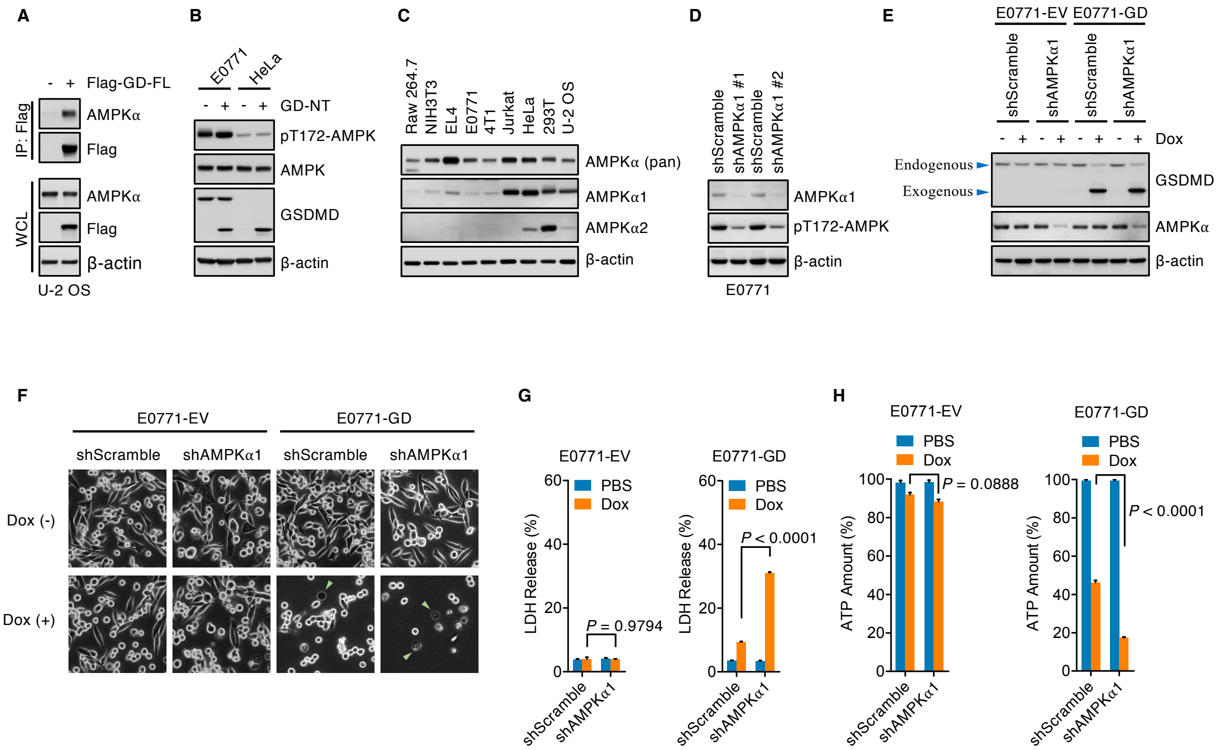

Extended Data Fig. 2 l AMPK protects tumor cells from GD-NT-mediated pyroptosis.

A, GD-NT interacts with AMPKα. U-2 OS cells were transiently transfected with Flag-GD-FL. 24 hours later, cells were harvested for anti-Flag IP assay. The precipitants and WCL were immunoblotted with indicated antibodies.

B, AMPK activation in E0771 cells and HeLa cells. Cells expressing Dox-inducible GD-NT were treated with or without Dox and harvested for IB analysis with indicated antibodies.

C, AMPK expression in tumor cells was assessed with IB analysis with indicated antibodies.

D, Knockdown efficiency of shAMPKα1 in E0771 cells. E0771 cells was transduced to express shAMPKα1 or scramble shRNA and subjected to IB analysis with indicated antibodies.

E-H, AMPK Inactivation sensitizes tumor cells for GD-NT-mediated pyroptosis. E0771 cells were modified to express Dox-inducible GD-NT and/or shAMPKα1 #1. Cells were treated with Dox (2 μg/ml) for 24 hours to induce the expression of GD-NT. Shown are IB analysis of WCL with indicated antibodies (E), phase-contrast images (F), cell death assessment with LDH-based Cytotoxicity Assay (G) and cell survival assessment with ATP-based cell (H).

In G and H, differences among groups were analyzed by two-tailed Student`s *t*-test (means ± s.e.m). Error bars represent variation range of duplicated experiments. Data are representative of at least two independent experiments.

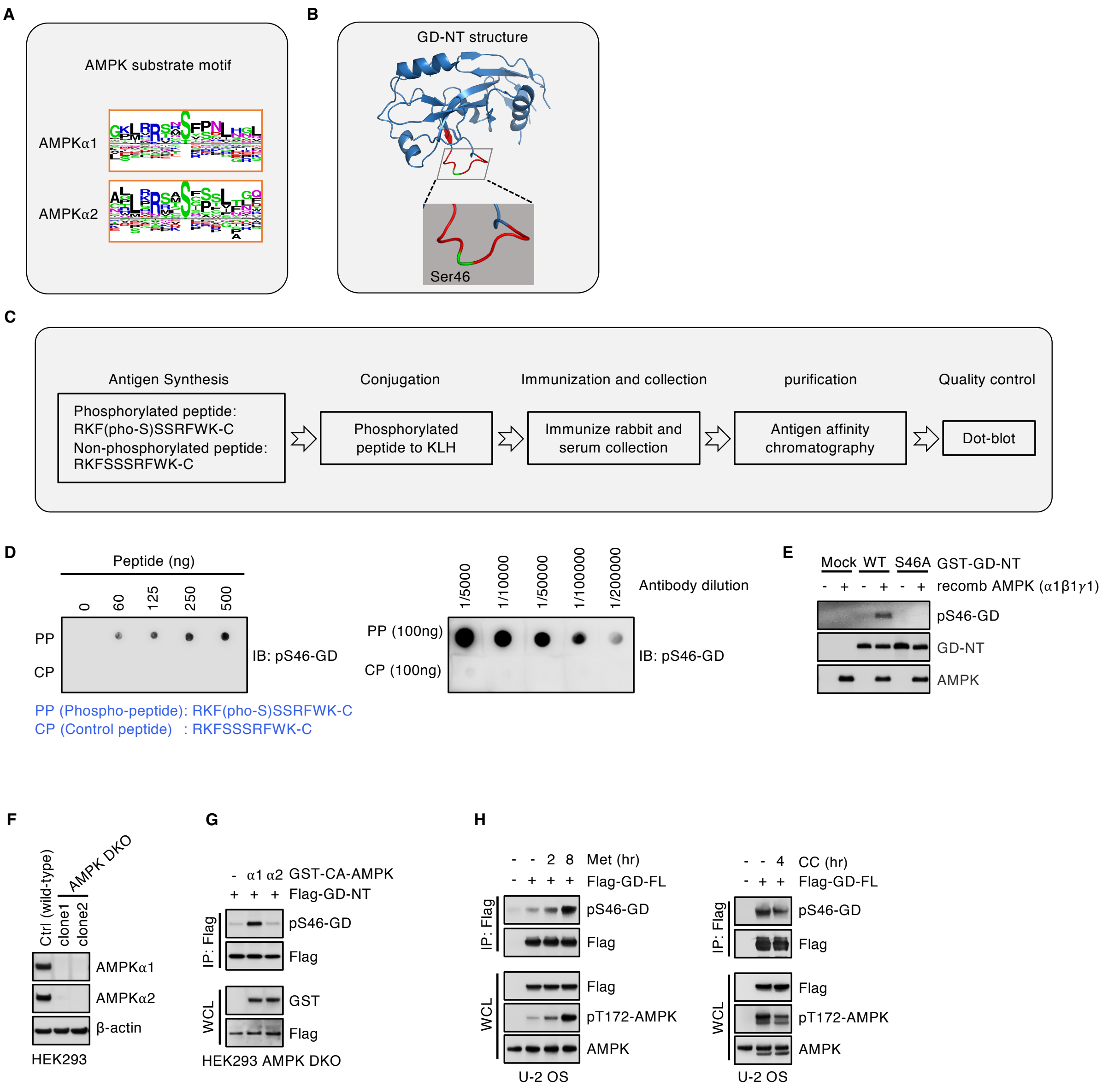

Extended Data Fig. 3 I AMPK targets GSDMD-Ser46.

A, AMPK phosphorylation consensus motif (L/M) XRXX(S/T) XXXL. Shown is AMPKα1 or AMPKα2 Substrate Sequence Logo. In the logo, the bigger font size indicates the preferred amino acid for AMPK targeting. Data was obtained from PhosphoSitePlus® and re-organized accordingly. PhosphoSitePlus® provides comprehensive information of protein post-translational modifications (www.phosphosite.org).

B, Ser46 locates in β1-β2 loops of GD-NT. Structure of mouse GD-NT was downloaded from RCSB PDB (PDB code: 6N9N). PyMOL was used to generate structure model of mouse GD-NT and label amino acid residues. The β1-β2 loops was colored in red and Ser46 was colored in green.

C, Design and generation of the specific antibody against phosphorylated Ser46 of mouse GSDMD.

D, Quality control of the antibody against pS46-GD via dot-blot. The modified and non-modified polypeptides were immobilized on Nitrocellulose membrane and subjected to immunoblot with the antibody against pS46-GD.

E, Similar to (Fig. 4E), except that *In vitro* kinase assay was performed using a commercial recombinant AMPK as the phosphorylation kinase.

F, Generation of AMPKα1/α2 double knockout cell clones (AMPK DKO) using CRISPR/Cas9 technology. Shown is IB analysis of HEK293 parental cells and AMPK DKO cell clones using the antibody specifically against AMPKα1 or AMPKα2. **G**, HEK293 AMPK DKO cells were co-transfected with Flag-GD-NT and GST-CA-AMPKα1 or AMPKα2 and subjected to anti-Flag IP assay. The precipitants and WCL were immunoblotted with indicated antibodies.

H, U-2 OS cells were transfected with Flag-GD-FL or empty vector. 24 hours later, cells were treated with metformin (10 mM) or Compound C (5 µM) for the indicated time and then harvested for anti-Flag IP assay. The precipitants and WCL were immunoblotted with indicated antibodies.

Data are representative of at least two independent experiments.

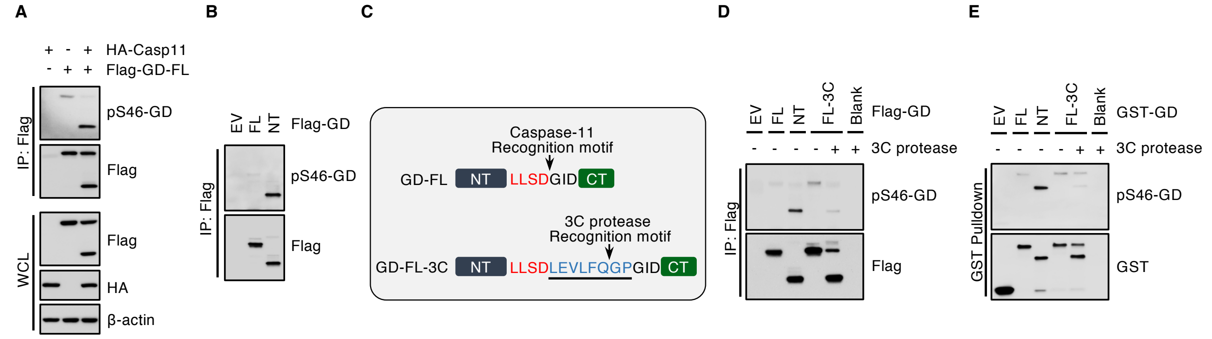

Extended Data Fig. 4 I GD-NT is more likely to undergo phosphorylation than GD-FL.

A, Flag-GD-FL were co-transfected with HA-caspase11 (Casp11) or empty vector in HEK293 cells. 16 hours later, cells were harvested for anti-Flag IP assay. The precipitants and WCL were immunoblotted with indicated antibodies.

B, HEK293 cells were transfected with empty vector, Flag-GD-FL or GD-NT. 16 hours later, cells were harvested for anti-Flag IP assay. The precipitants and WCL were immunoblotted with indicated antibodies.

C, Schematic illustration of GD-FL and its mutant 3C (GD-FL-3C). The mutant 3C is created by replacing the caspase-11 recognition sequence (LLSDGID) with 3C protease recognition motif (LLSDLEVLFQGPGID).

D, HEK293 cells were transfected with empty vector, Flag-GD-NT, GD-FL or FL-3C. 16 hours later, cells were harvested for anti-Flag IP assay. The precipitants were incubated with or without protease 3C for 20 minutes and then immunoblotted with indicated antibodies.

E, Similar to (d), except that the cells were transfected with GST-GD-NT, GD-FL, GD-FL-3C. Glutathione pulldown was performed instead of anti-Flag IP assay. The GST precipitants were incubated with or without protease 3C for 1 hour and then immunoblotted with indicated antibodies. Data are representative of at least two independent experiments.

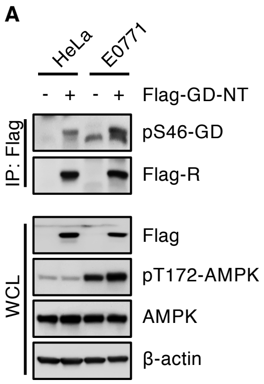

Extended Data Fig. 5 l GD-NT in HeLa cells is less phosphorylated than that of E0771 cells.

A, The pS46-GD level in HeLa cells and E0771 cells expressing Dox-inducible Flag-GD-NT. Cells were treated with or without Dox (2 μg/ml) for 16 hours and subjected to anti-Flag-IP assay. The precipitants and WCL were immunoblotted with indicated antibodies. Data are representative of at least two independent experiments.

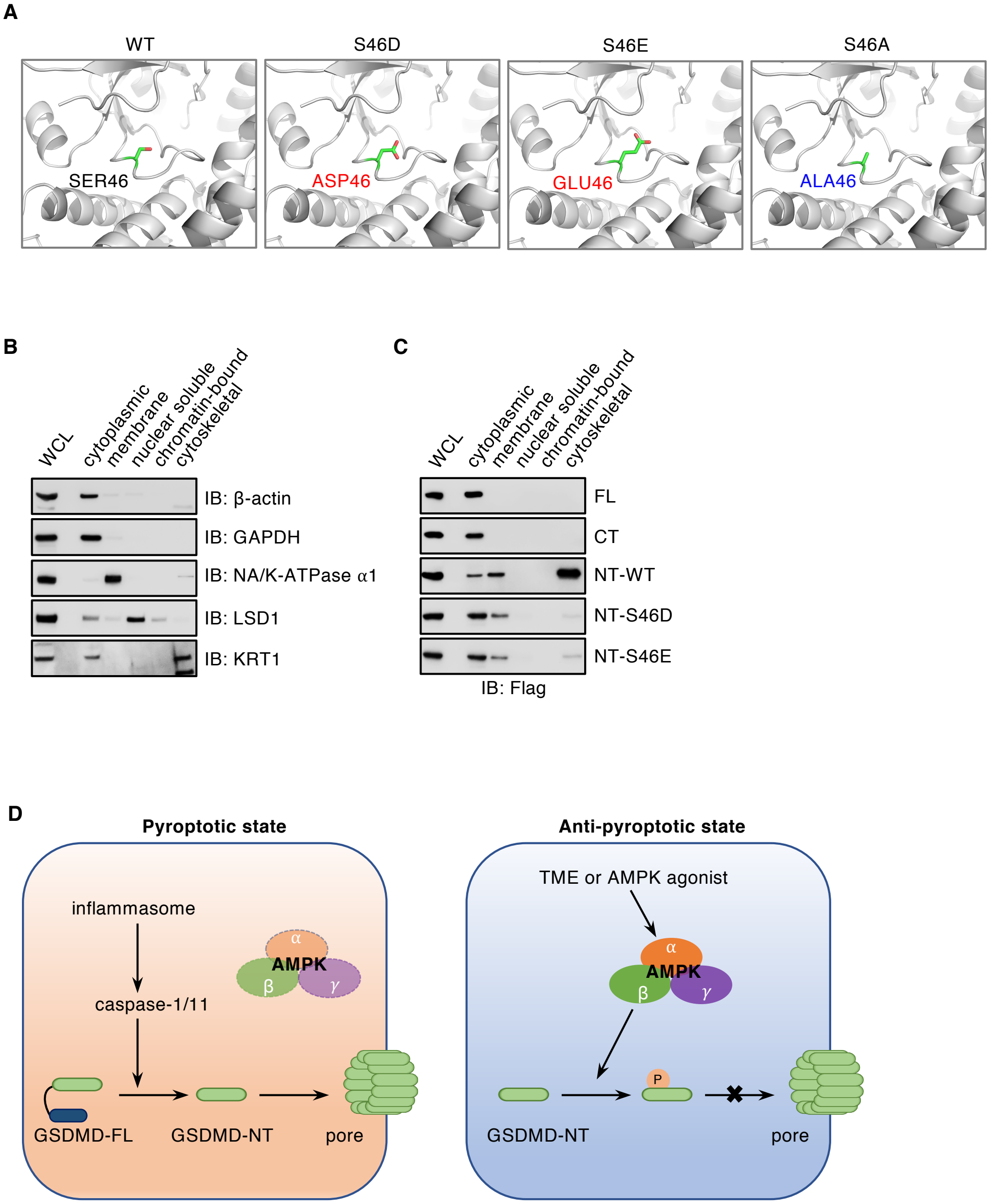

Extended Data Fig. 6 I Phosphorylation blocks GD-NT translocation to the plasma membrane.
A, Structural damage analysis of GSDMD site mutation. Structural damage was assessed with the online tool Missense3D. No structural damage was detected during the creation of GD-NT mutants via site-directed mutagenesis.
B, Subcellular Fractionation. HEK293 cells were fractionated using Thermo Fisher Subcellular Fractionation Kit and immunoblotted with indicated antibodies. The proteins with clear subcellular distribution, including β-actin, GAPDH, NA/K-ATPase α1, LSD1

and KRT1, were used as fractionation indicators. 10% WCL was included as input control.

C, Subcellular localization of GD-NT. Similar to (B), except that HEK293 cells were transfected with Flag-GD-FL, GD-CT, GD-NT-WT or its mutants. Protein fractions were immunoblotted with anti-Flag antibody.

D, Schematic illustration of AMPK-mediated inactivation of GD-NT. Pyroptotic stimuli trigger the assembly of inflammasome and maturation of caspase-1/11. The mature caspase-1/11 cleaves GD-FL to produce the pore-forming fragment GD-NT. In the scenarios of AMPK activation, GD-NT is phosphorylated by AMPK and loses pore-forming activity. In B and C, data are representative of at least two independent experiments.

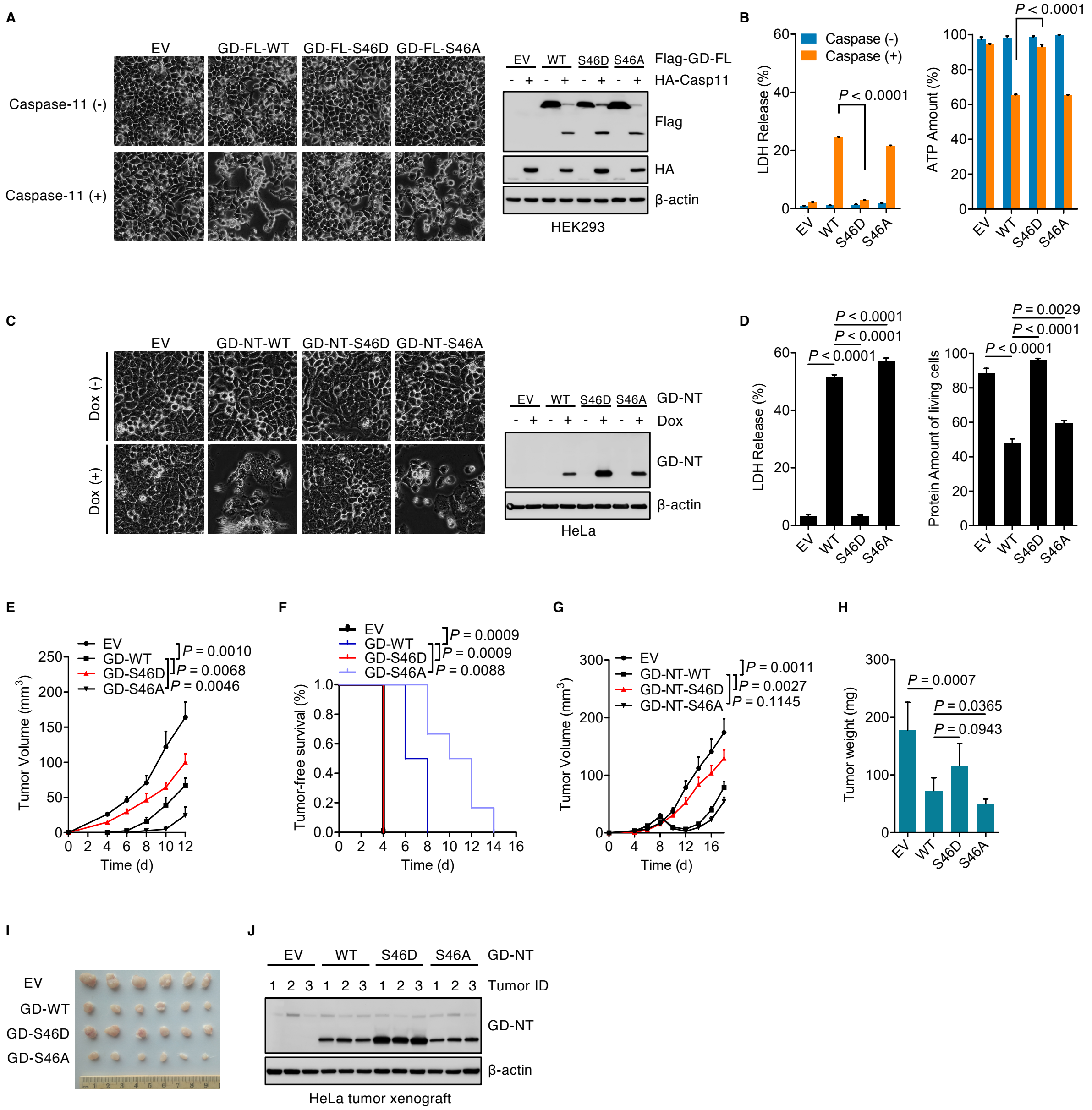

Extended Data Fig. 7 I Phosphorylated GD-NT loses the ability to mediate pyroptosis both in *vitro* and in *vivo*.

A, HEK293 cells were transfected with HA-caspase11 and Flag-GD-FL-WT or its mutants. 24 hours later, cells were harvested for further analysis. Shown are phase-contrast images (left panel) and IB analysis of WCL with indicated antibodies (right panel).

B, Similar to (A), except that the cells were harvested and subjected to LDH-based Cytotoxicity Assay or ATP-based Cell Viability Assay.

C, HeLa cells expressing Dox-inducible GD-NT-WT or its mutants were treated with or without Dox (2 μg/ml). 48 hours later, cells were harvested for further analysis. Shown

are phase-contrast images (left panel) and IB analysis of WCL with indicated antibodies (right panel).

D, Similar to (C), except that the cells were subjected to cell death assessment using LDH-based Cytotoxicity Assay or BCA protein quantification.

E-F, HeLa tumor xenograft in NSG mice. HeLa cells expressing Dox-inducible GD-NT-WT or its mutants (0.5 x 10^6 cells per mouse) were subcutaneously implanted into the right flank of NSG mice (n = 6 mice per group). Dox (50 mg/kg, i.p.) was administrated on d2 and the following every other day. Tumor growth was recorded every other day. Shown are tumor growth (E) and tumor-free survival (F).

G-J, HeLa tumor xenograft in NSG mice. Similar to (E-F), except that Dox (50 mg/kg, i.p.) was administrated on d6 and the following every other day. Tumor growth was recorded every other day. Shown are tumor growth (G), tumor weight (H), tumor images (I) and IB analysis of 3 tumor tissues randomly taken from each group (J).

In B, D, E, G and H, error bars represent variation range of duplicated experiments. In B, D and H, differences among groups were analyzed by two-tailed Student`s *t*-test (means ± s.e.m). In E and G, the areas under the growth curves were compared by two-tailed Student`s *t*-test (means ± s.e.m). In F, a log-rank test was used for tumor-free survival. Data are representative of at least two independent experiments.

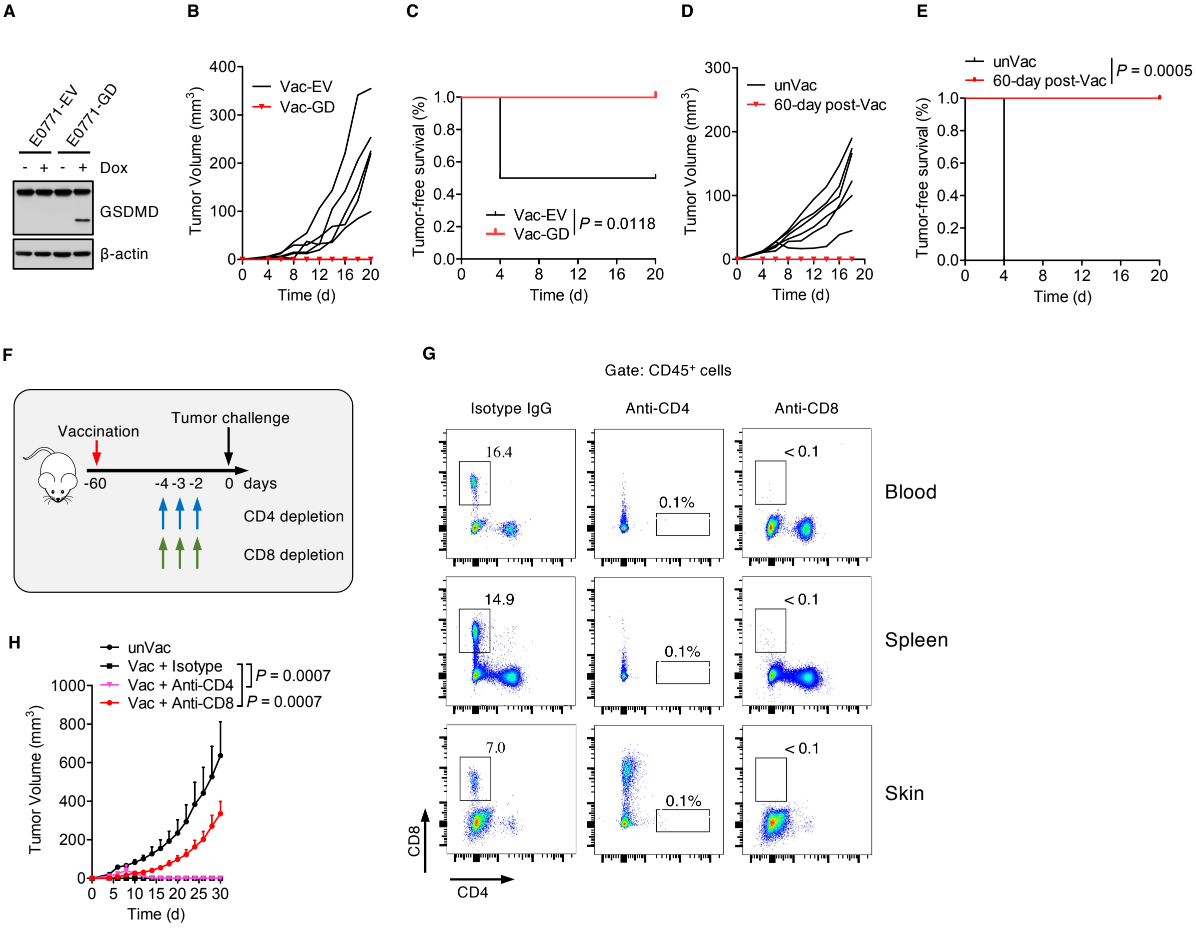

Extended Data Fig. 8 I GD-NT-based whole-cell vaccine induces anti-tumor immunity.

A, E0771 cells were transduced to stably express Dox-inducible GD-NT (E0771-GD) or empty vector (E0771-EV). The ectopic expression of GD-NT was assessed by immunoblot with indicated antibodies.

B-C, GD-NT triggers anti-tumor immunity. C57BL/6 naïve mice were vaccinated with 1.0 x 10^6 of E0771-EV cells (named Vac-EV mice) or E0771-GD-NT cells (named Vac-GD mice) in the left fourth mammary fat pad (n = 10 mice per group). 14 days post-vaccination, mice were challenged with 1.0 x 10^6 of E0771 parental cells in the right fourth mammary fat pad. Shown are tumor growth curves (B) and tumor-free survival (C).

D-E, Vac-GD mice acquire long-term anti-tumor memory. Vac-GD mice or Naïve mice were challenged with 1.0 x 10^6 of E0771 parental cells on d60 post-vaccination (n = 6 mice per group). Shown are tumor growth curves (D) and tumor-free survival (E).

F-H, CD8+ T cells contribute to GD-NT-induced anti-tumor immunity. Vac-GD mice were intraperitoneally injected with anti-CD4, anti-CD8α or isotype control antibody and then were challenged with 1.0 x 10^6 of E0771 parental cells as shown (n=6 mice per group) (F). Depletion efficiency of T cell subsets was assessed on day 0 by FCAS analysis (G), and tumor growth curves were recorded as shown (H).

In C and E, a log-rank test was used for tumor-free survival. In H, the areas under the growth curves were compared by two-tailed Student`s *t*-test (means ± s.e.m). Error bars represent variation range of duplicated experiments. Data are representative of at least two independent experiments.

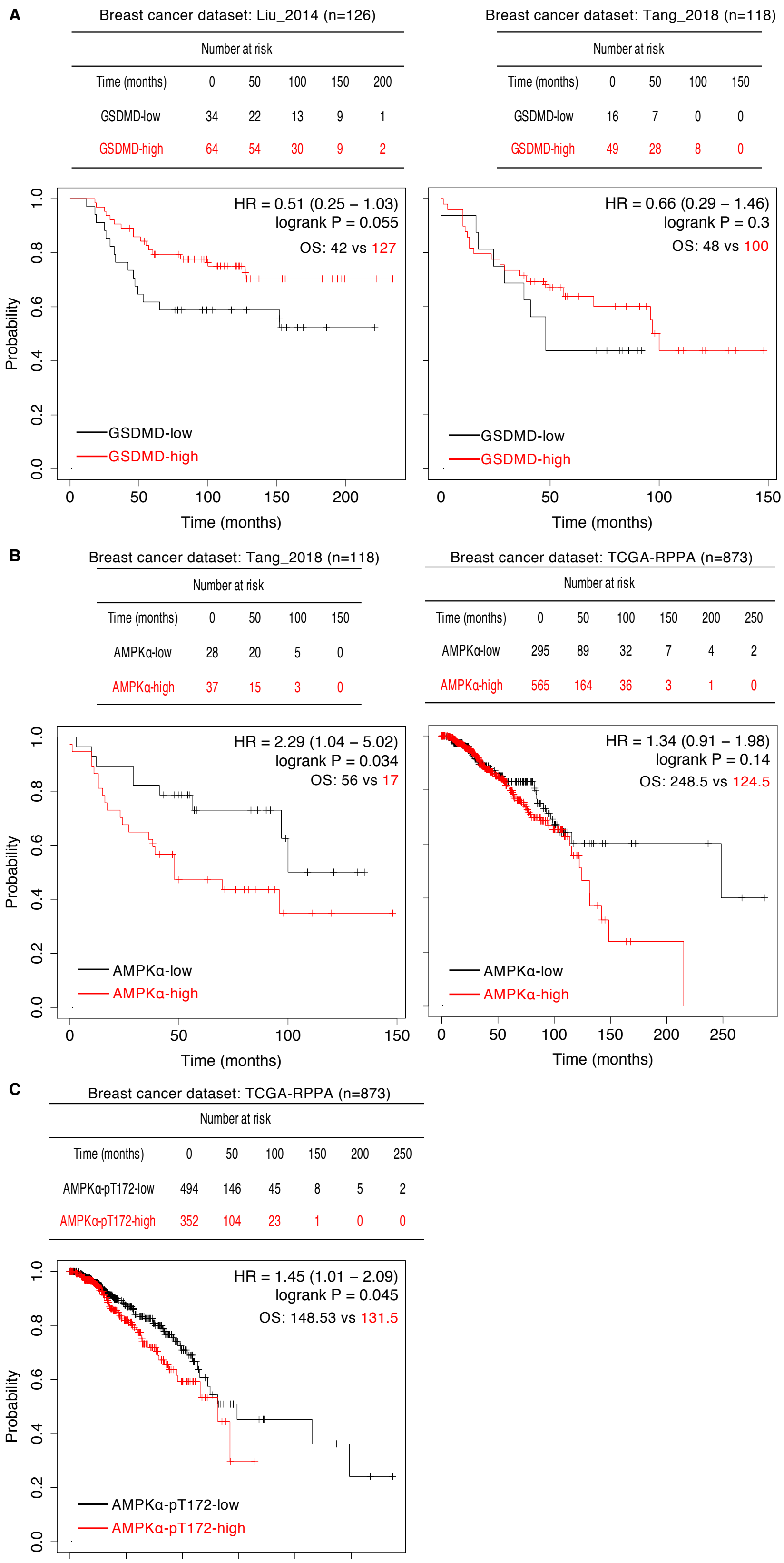
A
Breast cancer dataset: Liu_2014 (n=126)
Number at risk
Time (months) 0 50 100 150 200
GSDMD-low 34 22 13 9 1
GSDMD-high 64 54 30 9 2
HR = 0.51 (0.25 – 1.03)
logrank P = 0.055
OS: 42 vs 127
GSDMD-low
GSDMD-high
Probability
Time (months)
Breast cancer dataset: Tang_2018 (n=118)
Number at risk
Time (months) 0 50 100 150
GSDMD-low 16 7 0 0
GSDMD-high 49 28 8 0
HR = 0.66 (0.29 – 1.46)
logrank P = 0.3
OS: 48 vs 100
GSDMD-low
GSDMD-high
Time (months)
B
Breast cancer dataset: Tang_2018 (n=118)
Number at risk
Time (months) 0 50 100 150
AMPKα-low 28 20 5 0
AMPKα-high 37 15 3 0
HR = 2.29 (1.04 – 5.02)
logrank P = 0.034
OS: 56 vs 17
AMPKα-low
AMPKα-high
Probability
Time (months)
Breast cancer dataset: TCGA-RPPA (n=873)
Number at risk
Time (months) 0 50 100 150 200 250
AMPKα-low 295 89 32 7 4 2
AMPKα-high 565 164 36 3 1 0
HR = 1.34 (0.91 – 1.98)
logrank P = 0.14
OS: 248.5 vs 124.5
AMPKα-low
AMPKα-high
Time (months)
C
Breast cancer dataset: TCGA-RPPA (n=873)
Number at risk
Time (months) 0 50 100 150 200 250
AMPKα-pT172-low 494 146 45 8 5 2
AMPKα-pT172-high 352 104 23 1 0 0
HR = 1.45 (1.01 – 2.09)
logrank P = 0.045
OS: 148.53 vs 131.5
AMPKα-pT172-low
AMPKα-pT172-high
Probability

Extended Data Fig. 9 | Survival analysis in breast cancer using proteomic datasets.

A, Association between GSDMD expression and patient survival. Patient Data were taken from dataset Liu_2014 (PXD000260, n=126) and Tang_2018 (PXD005692, n=118). The correlation between GSDMD expression and survival was assessed using Kaplan Meier plotter (https://kmplot.com/analysis/). Briefly, the patients were grouped into high and low expression groups based on the expression of GSDMD. The two groups were compared by Cox proportional hazards regression. Hazard ratio (HR), 95% confidence interval, log-rank *p* values were calculated. Kaplan-Meier plot was generated to display the different survival characteristics of the two groups. Of note, the Liu_2014 dataset included triple negative breast cancer, lymph node negative and treatment naïve patients only.

B, Similar to (A), except that the correlation between AMPKα and survival length was assessed and patient data were taken from dataset Tang_2018 (PXD005692, n=118) and TCGA-RPPA (n=873).

C, Similar to (A), except that the correlation between AMPKα-pT172 and survival length was assessed and patient data were taken from dataset TCGA-RPPA (n=873).
