# Supplementary material for: Gasdermin D-mediated pyroptosis is regulated by AMPK-mediated phosphorylation in tumor cells
Source: Cell Death Dis. 2023 Jul 26;14(7):469. doi: 10.1038/s41419-023-06013-6 (PMC10372026; doi:10.1038/s41419-023-06013-6)
Supplement: Supplementary file 3 — CDDis Uncropped original western blot [file 41419_2023_6013_MOESM3_ESM.pdf]

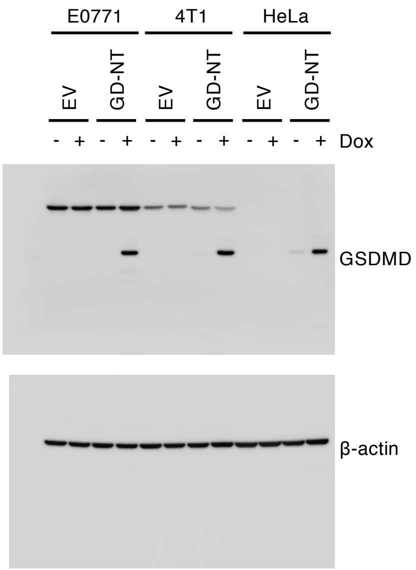

Fig. 1 | GD-NT-interacting kinases may regulate the resistance of tumor cells to GD-NT-mediated pyroptosis.

A

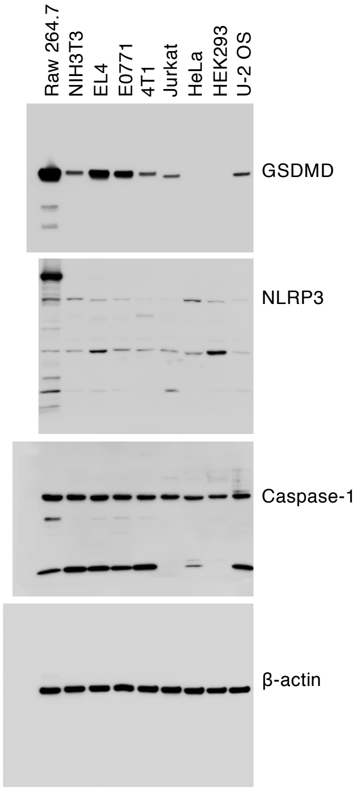

B

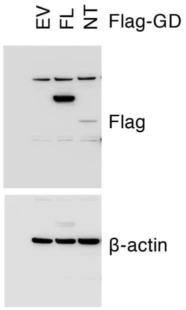

H

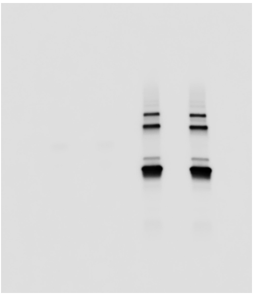

IB: Flag

Extended Data Fig. 1 | GD-NT mediates pyroptotic cell death.

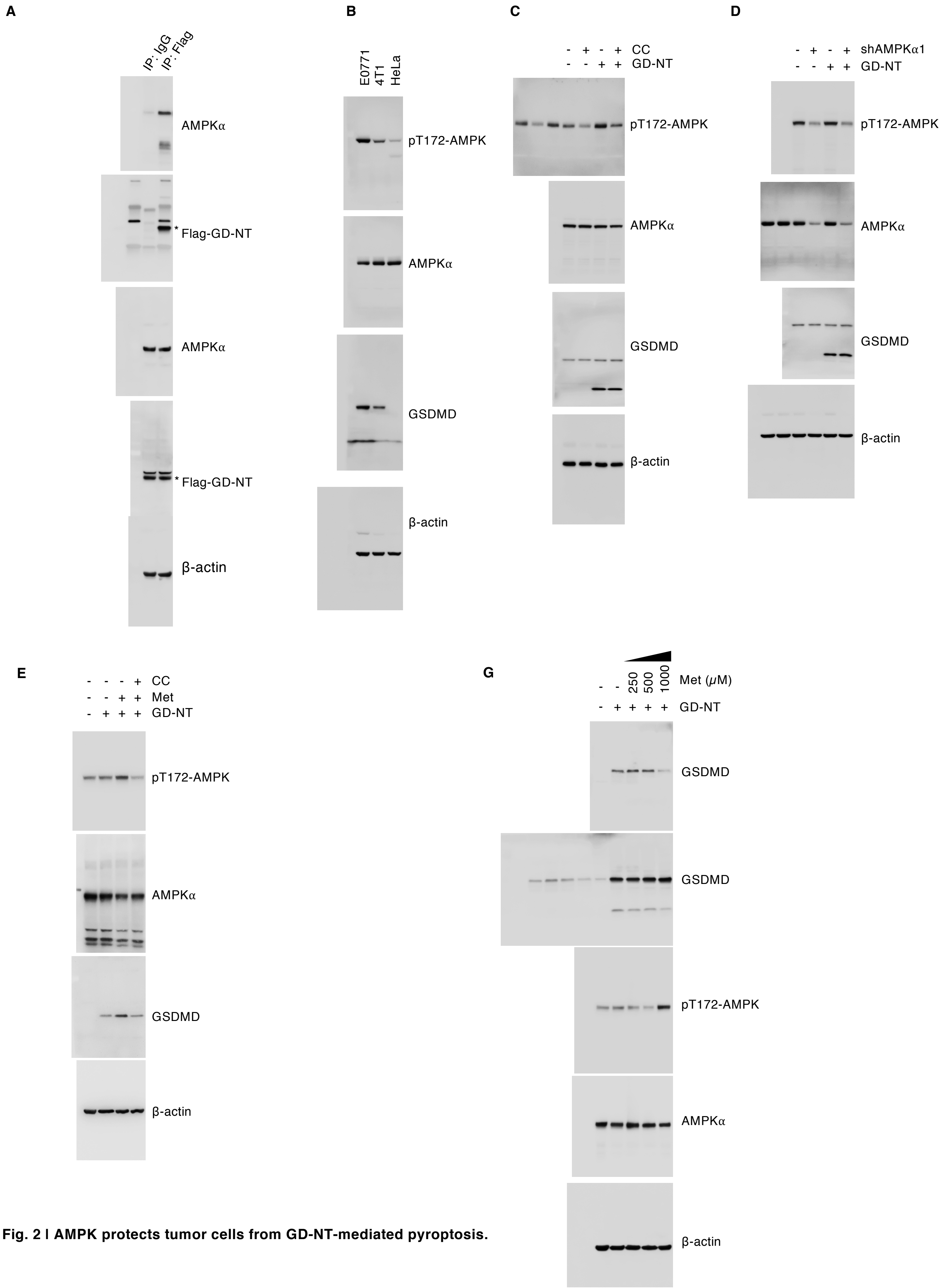

Fig. 2 | AMPK protects tumor cells from GD-NT-mediated pyroptosis.

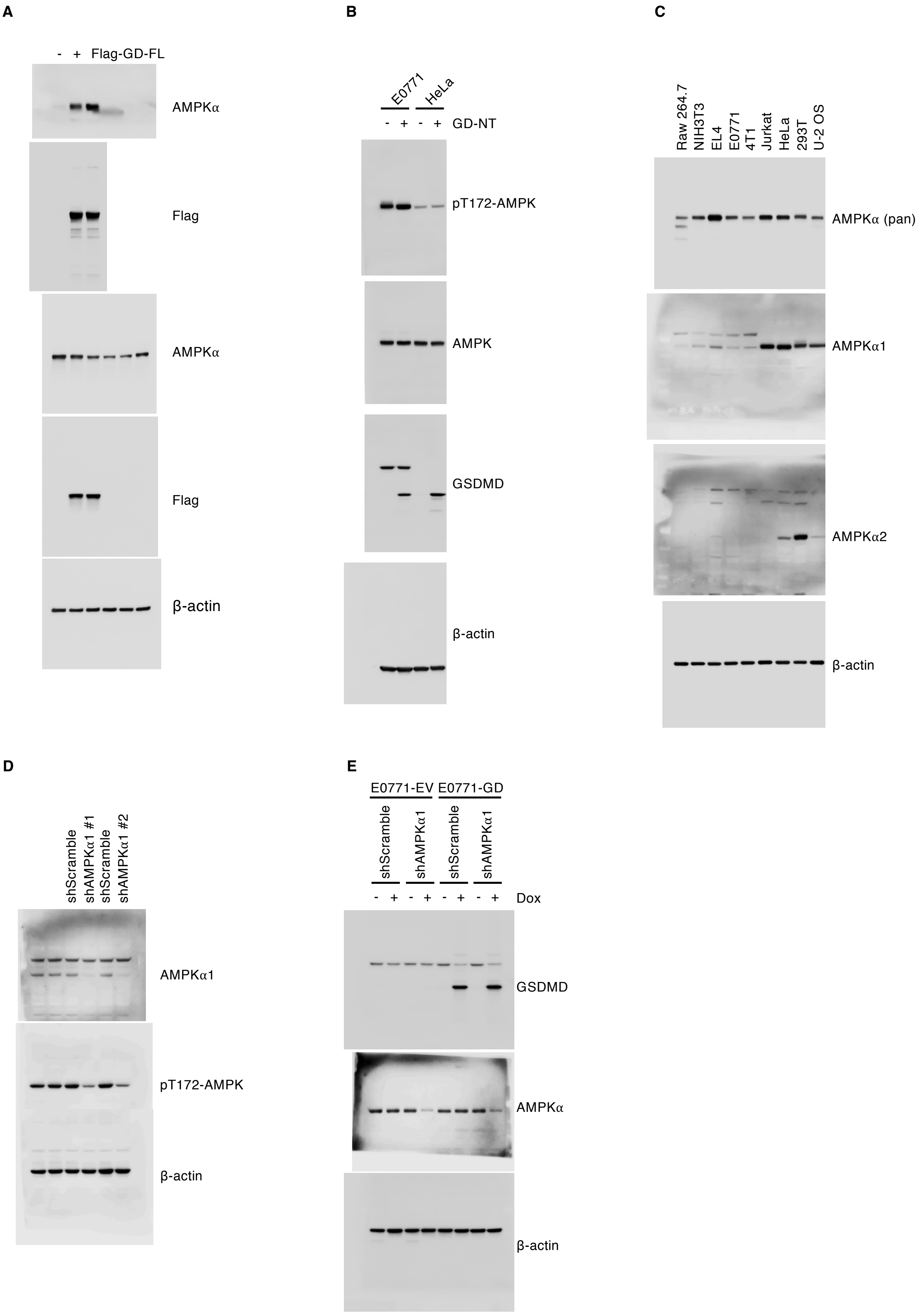

Extended Data Fig. 2 | AMPK protects tumor cells from GD-NT-mediated pyroptosis.

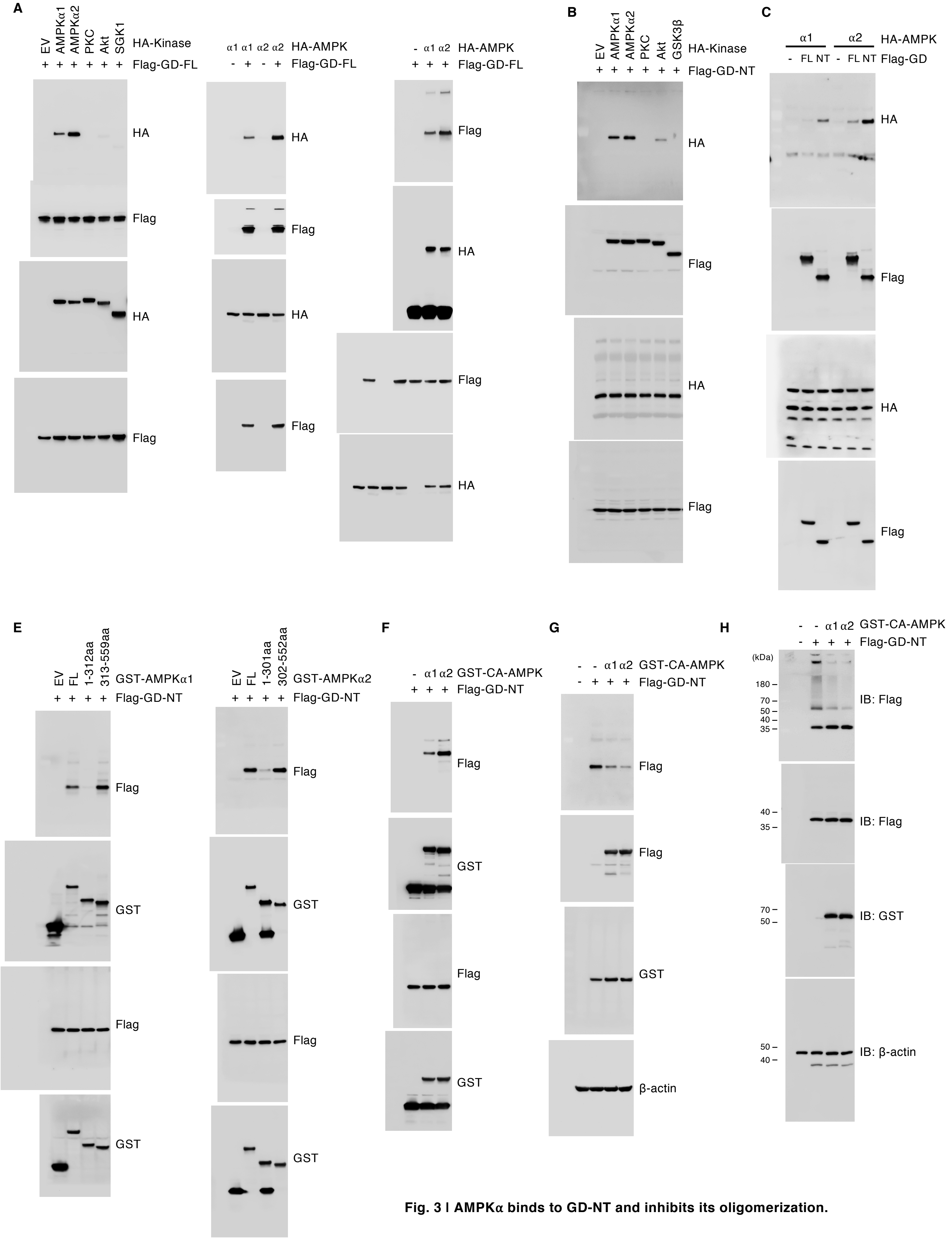

Fig. 3 | AMPKα binds to GD-NT and inhibits its oligomerization.

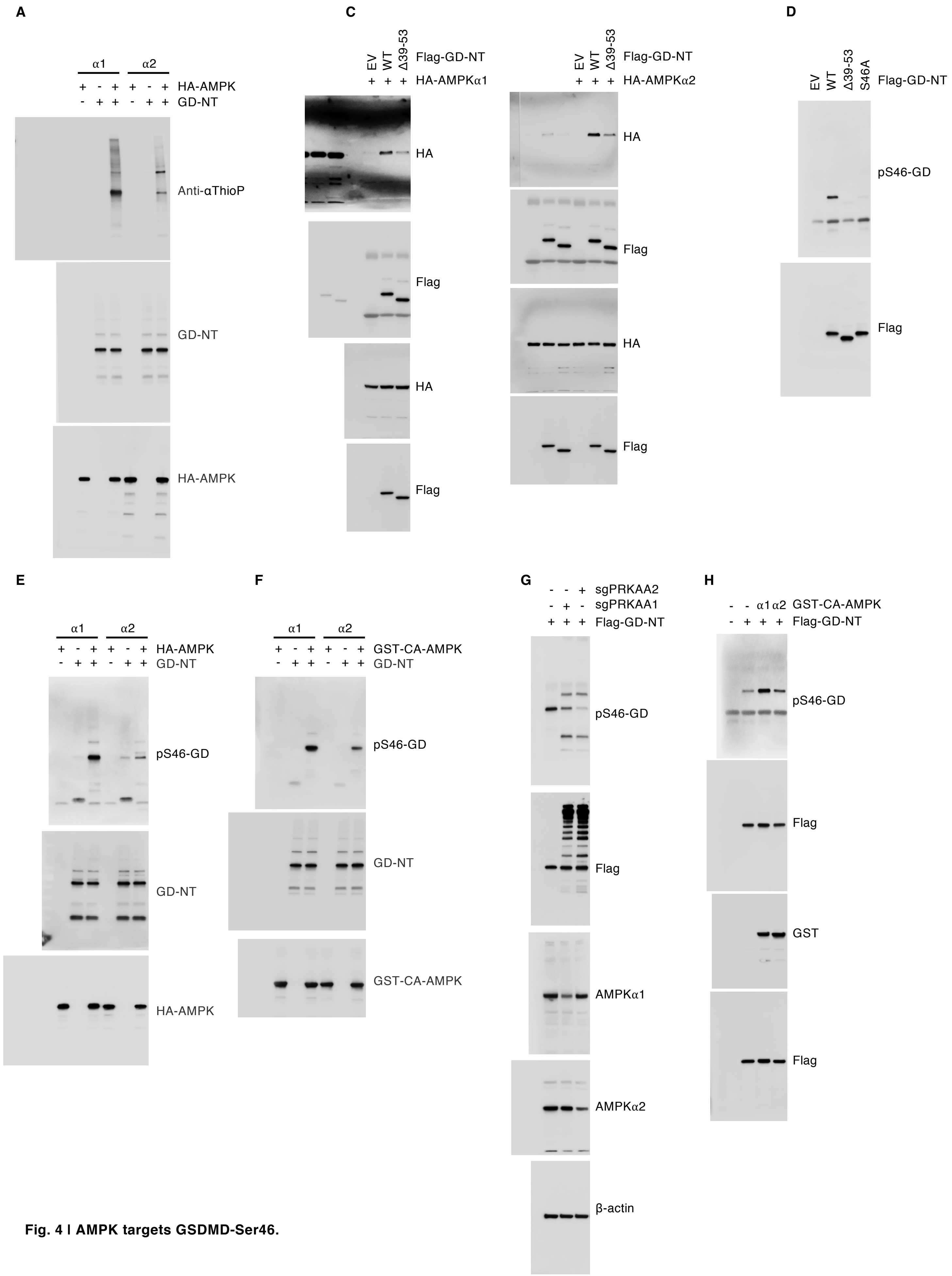

Fig. 4 | AMPK targets GSDMD-Ser46.

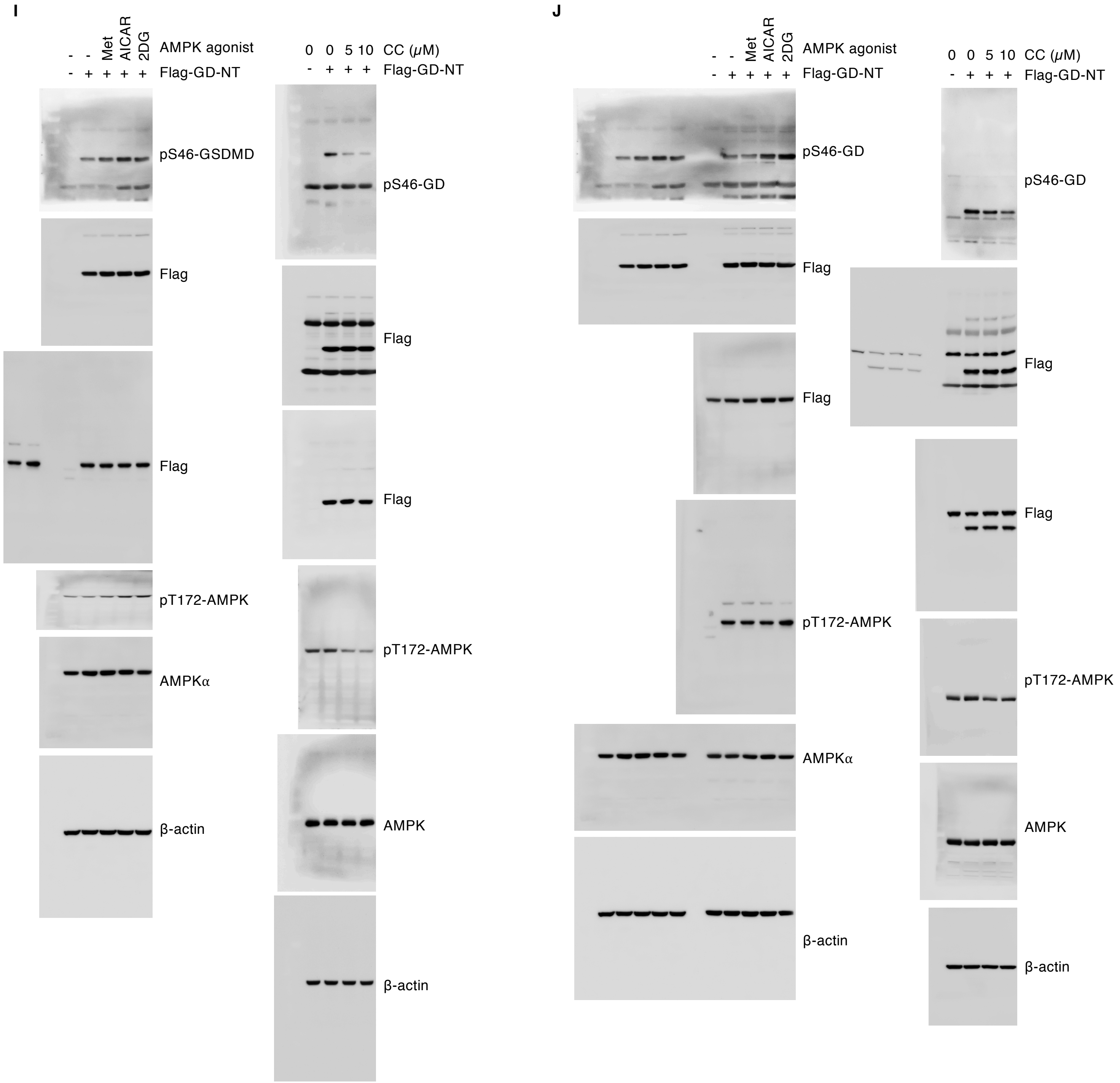

Fig. 4 | AMPK targets GSDMD-Ser46. (continued)

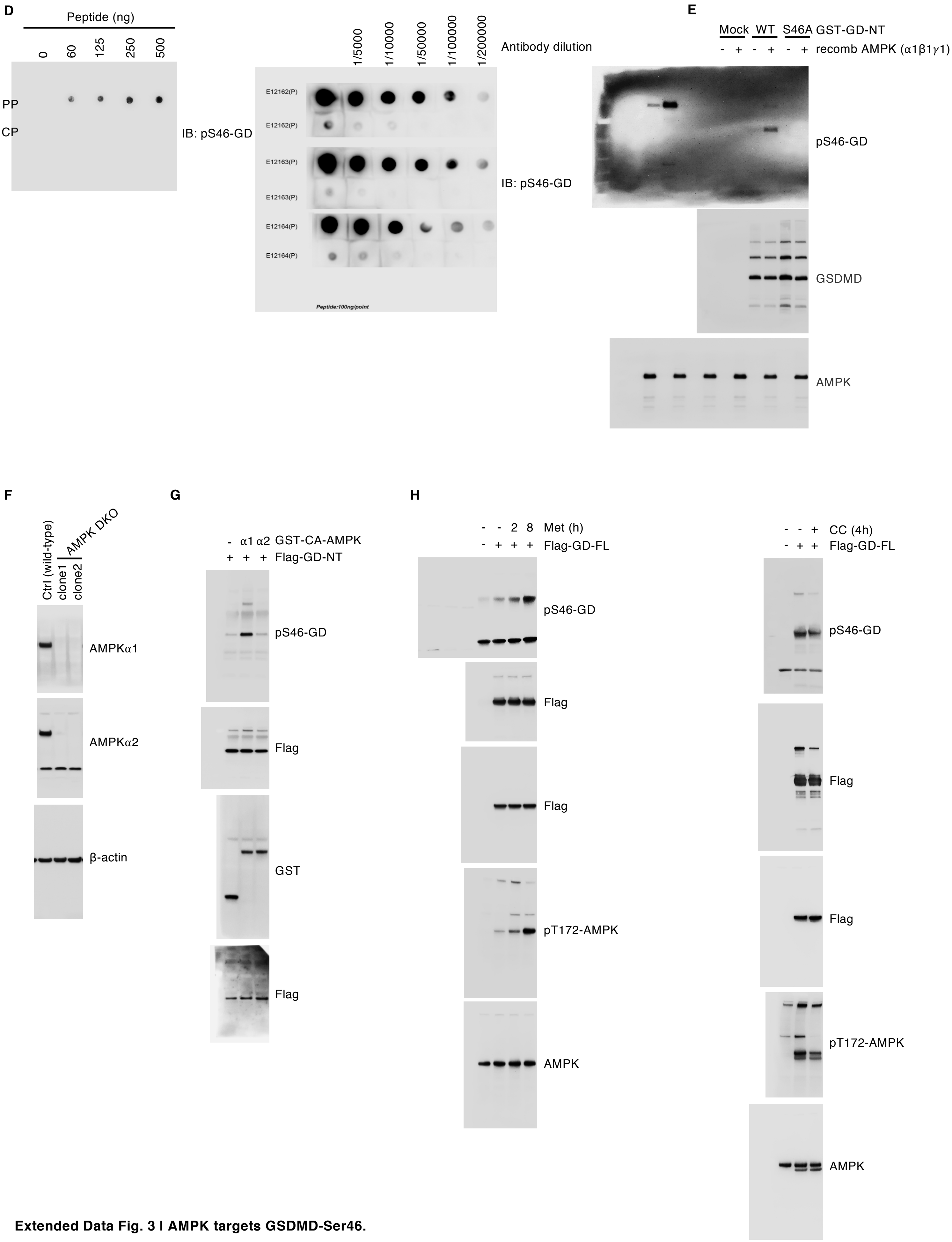

Extended Data Fig. 3 | AMPK targets GSDMD-Ser46.

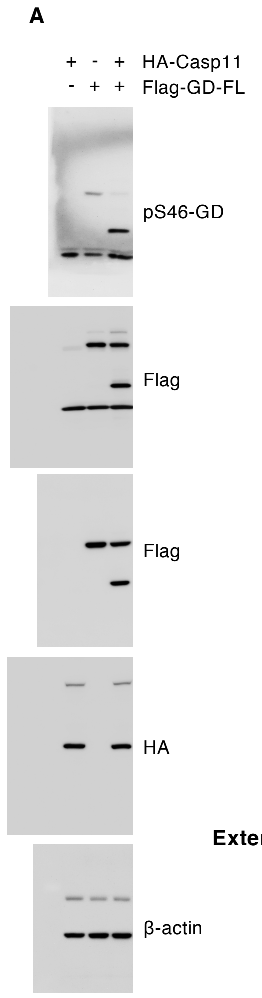

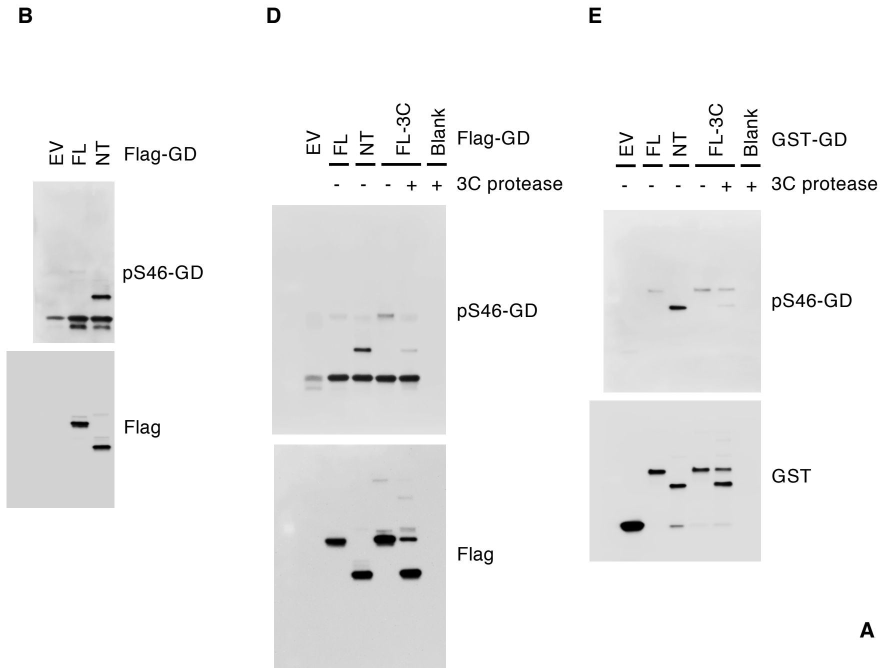

Extended Data Fig. 4 | GD-NT is more likely to undergo phosphorylation than GD-FL.

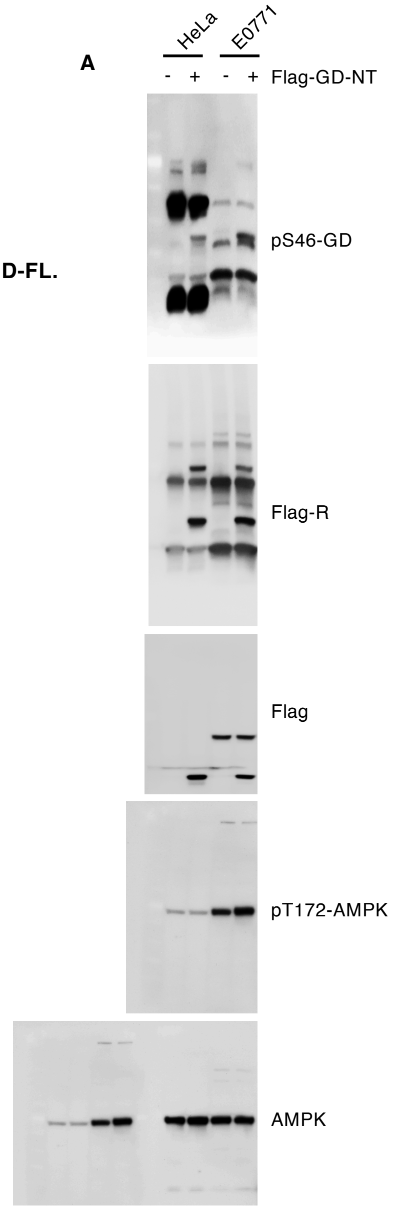

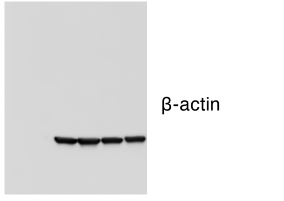

Extended Data Fig. 5 | GD-NT in HeLa cells is less phosphorylated than that of E0771 cells.

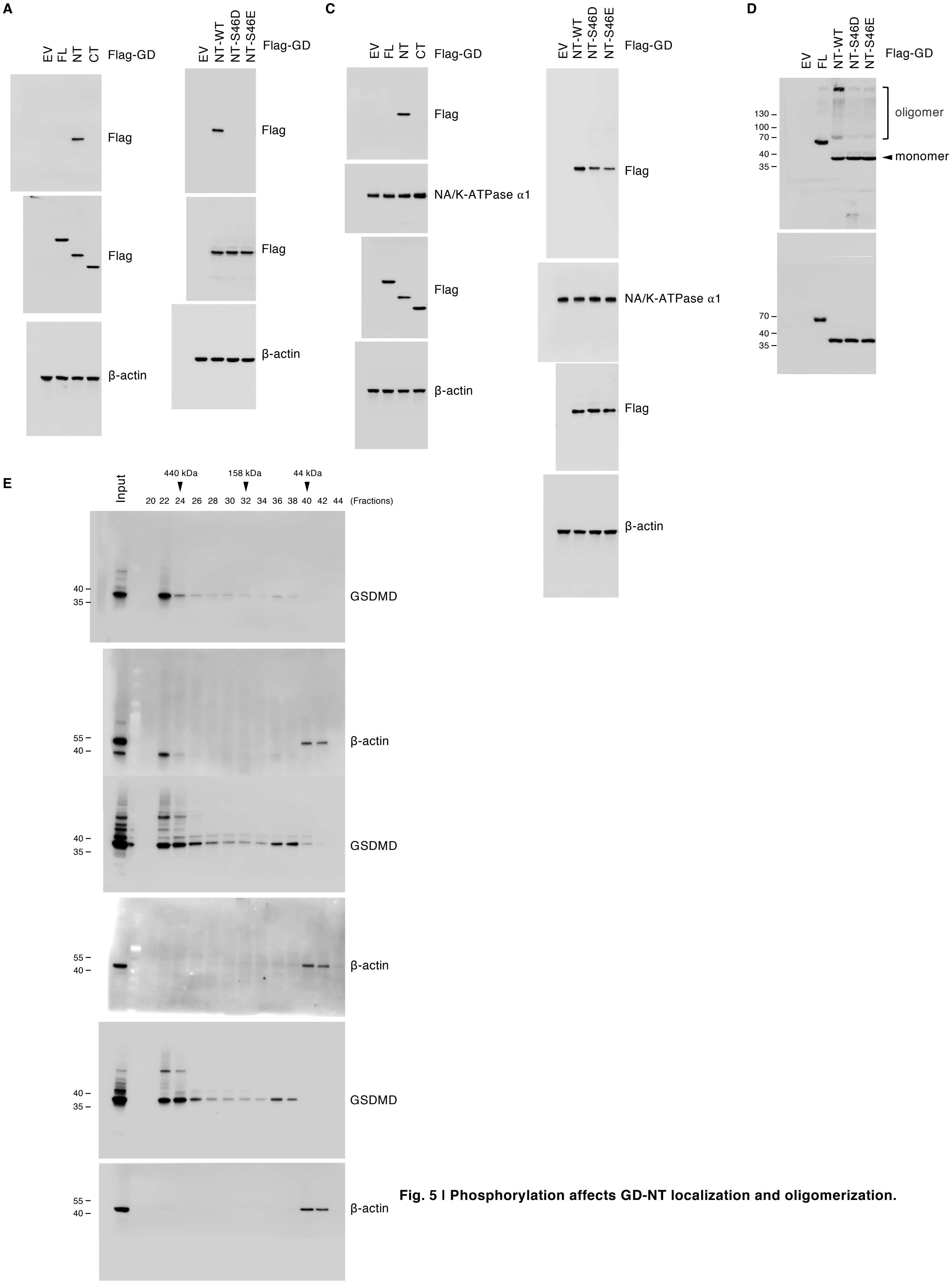

Fig. 5 | Phosphorylation affects GD-NT localization and oligomerization.

B

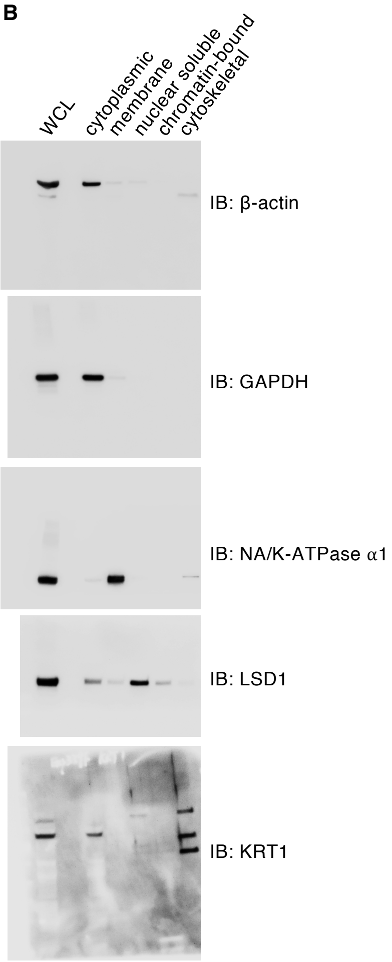

C

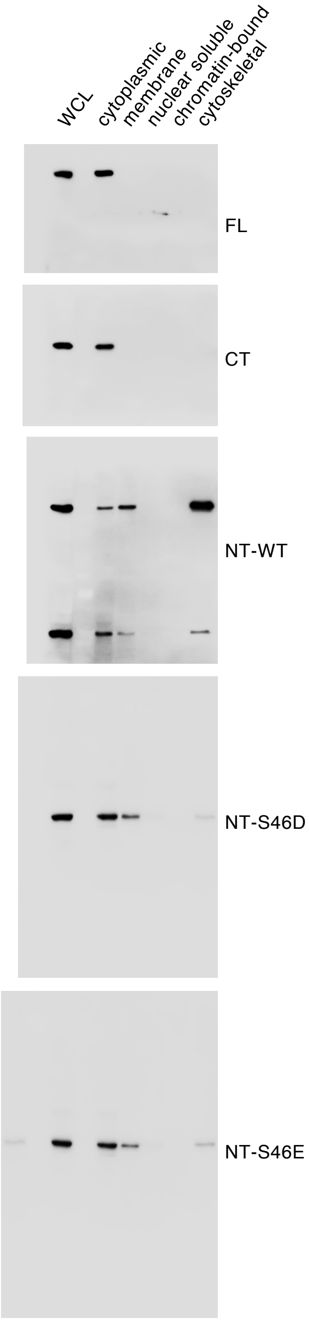

Extended Data Fig. 6 | Phosphorylation blocks GD-NT translocation to the plasma membrane.

A

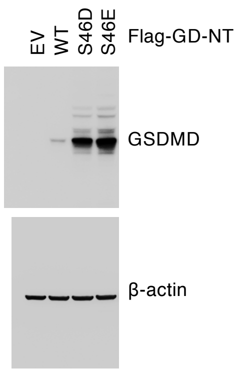

C

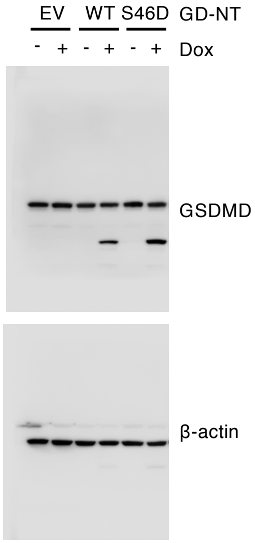

Fig. 6 | Phosphorylated GSDMD loses the ability to mediate pyroptosis and anti-tumor immunity *in vivo*.

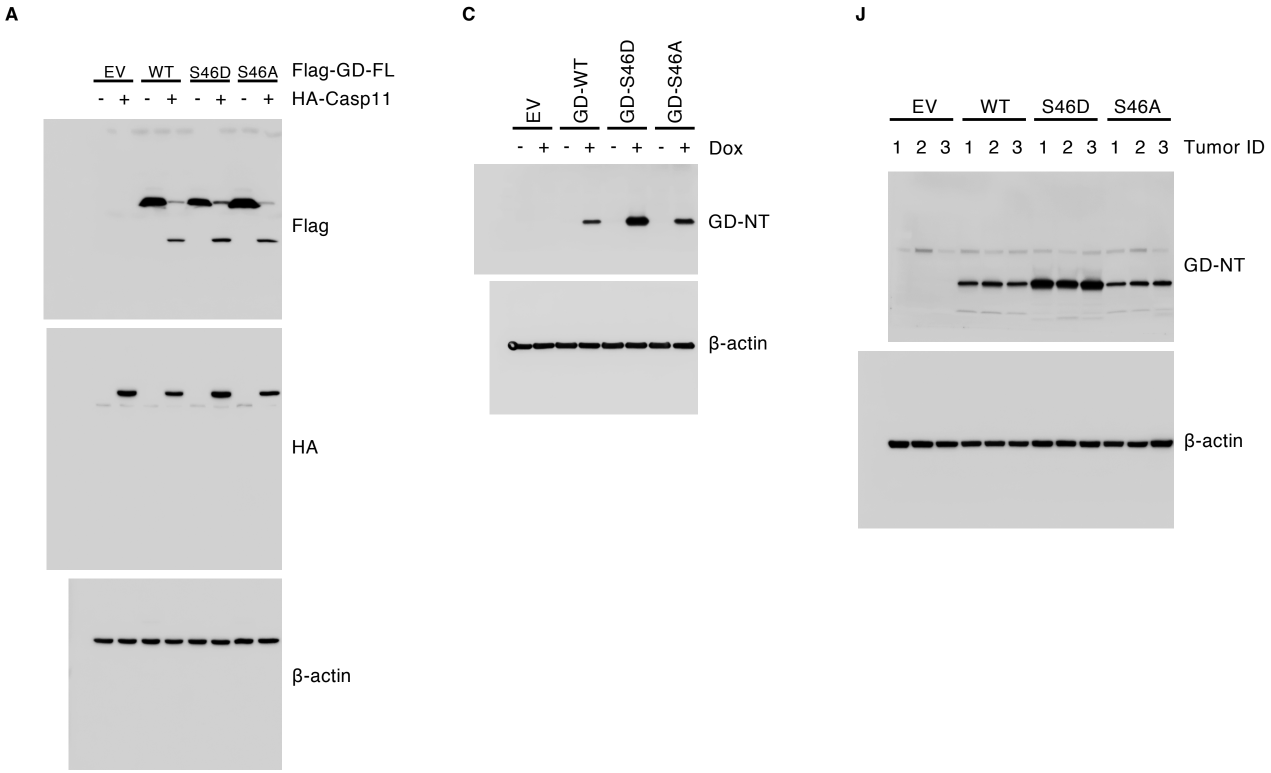

Extended Data Fig. 7 | Phosphorylated GD-NT loses the ability to mediate pyroptosis both in *vitro* and in *vivo*.

A

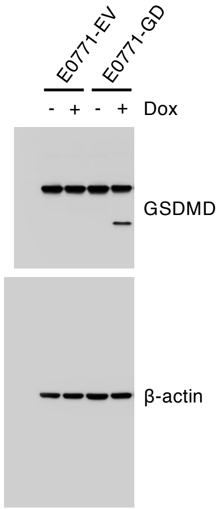

Extended Data Fig. 8 | GD-NT-based whole-cell vaccine induces anti-tumor immunity.
